# Evaluation of large language model responses to patient questions on oral anticoagulant therapy: a comparative expert assessment

**DOI:** 10.1016/j.rcsop.2026.100812

**Published:** 2026-06-09

**Authors:** Mohammed Amer Khan, Vaibhav Chaudhary, Mohammed Maazuddin, Ihtisham Sultan, Saamiya Mehnaaz, Biplab Pal

**Affiliations:** a School of Pharmaceutical Science, Lovely Professional University, Phagwara, Punjab, India; b Clinical Pharmacist, Jawaharlal Nehru Technological University Hyderabad, India; c Clinical Pharmacist, Sri Venkateswara College of Pharmacy, Osmania University, Hyderabad, India

**Keywords:** Large language models, Oral anticoagulants, ChatGPT, Gemini, DeepSeek, Patient education

## Abstract

**Background:**

Large language model (LLM)–based chatbots are increasingly used by patients for health information; however, their reliability in high-risk cardiovascular therapies such as oral anticoagulation remains uncertain. This study evaluated the perceived accuracy, clarity, and completeness of LLM-generated responses to common patient queries compared with standard-derived expert responses (SDERs).

**Methods:**

A cross-sectional comparative study evaluated responses generated by ChatGPT-4.5, Gemini Pro 2.5, and DeepSeek-V3 to 11 frequently asked questions related to five oral anticoagulants: warfarin, dabigatran, apixaban, rivaroxaban, and edoxaban. Responses were generated using a standardized patient-focused prompt. SDERs were developed by cardiologists and clinical pharmacists using authoritative references. All responses were anonymized and independently assessed by two blinded clinical pharmacists using a five-point Likert scale evaluating accuracy, clarity, and completeness. Interrater reliability was assessed using linearly weighted Cohen's κ, and group comparisons were analyzed using the Friedman test with post hoc adjustments.

**Results:**

Interrater reliability ranged from fair to almost perfect (κ = 0.31–0.84). ChatGPT-4.5 achieved the highest mean ratings across all evaluation domains, particularly for completeness. Significant differences were observed among response sources for accuracy, clarity, and completeness (*p* < 0.05). Warfarin-related queries demonstrated significant differences in accuracy and completeness across response sources, whereas responses for direct oral anticoagulants showed no significant differences.

**Conclusion:**

ChatGPT-4.5 received the highest mean expert ratings for patient education regarding oral anticoagulants. Performance differences were most evident for warfarin-related queries, whereas responses for direct oral anticoagulants were broadly comparable across sources.

## 1 Introduction

The emergence of large language model (LLM) chatbots has reshaped how health information is accessed and consumed. Since the launch of the Chat Generative Pretrained Transformer (ChatGPT), these tools have attracted substantial attention from both patients and healthcare professionals due to their potential to enhance communication and support clinical decision-making.^1^, ^2^, ^3^, ^4^, ^5^ This technological shift now extends beyond ChatGPT to other advanced LLMs, including Gemini and DeepSeek, which demonstrate varying levels of applicability within healthcare settings.

Recent studies have highlighted the potential of these models to generate clinically relevant patient education materials. Gemini has demonstrated the ability to provide accurate and comprehensive responses to oral cancer–related inquiries,^6^ while DeepSeek has shown utility in addressing patient questions regarding laparoscopic cholecystectomy.^7^ Collectively, this evidence suggests that LLMs can, in certain domains, generate medical information comparable in quality to that provided by healthcare professionals.^8^, ^9^ Nevertheless, concerns persist regarding the reliability of LLM-generated outputs in clinical areas where precision is critical. Oral anticoagulant therapy represents one such domain, given its narrow therapeutic index, substantial bleeding risk, complex drug–drug and drug–food interactions, and frequent requirement for individualized dosing.^10^, ^11^, ^12^ In this context, misinformation or incomplete patient education may result in serious clinical consequences, including thromboembolic events and major bleeding complications.

The urgency of addressing this issue is underscored by the growing global burden of anticoagulant use. The prevalence of atrial fibrillation (AF), the most common indication for oral anticoagulation, increased from 33.5 million to 59 million people between 2010 and 2019.^13^ This epidemiological trend highlights the pressing need for reliable, accessible, and patient-centered educational resources to support the safe and effective use of anticoagulant therapy.

Despite this need, current online health information resources frequently fail to meet clinical standards. Digital health content is often incomplete, inconsistent, or lacking sufficient clinical context.^14^ As patients increasingly turn to LLMs such as ChatGPT, Gemini, and DeepSeek for health-related information,^15^, ^16^, ^17^ their ability to deliver accurate, clear, and comprehensive education on oral anticoagulants has not yet been systematically evaluated.

To address this gap, the present study systematically evaluated the accuracy, clarity, and completeness of LLM-generated responses to frequently asked questions (FAQs) related to oral anticoagulants. By comparing responses generated by ChatGPT, Gemini, and DeepSeek with standard-derived expert responses (SDERs), this study aims to provide evidence-based insights into the quality of LLM-generated patient education on oral anticoagulation therapy. The findings seek to inform clinical practice, enhance patient safety, and support the development of reliable digital health resources that improve medication-related information and facilitate effective communication between patients and healthcare professionals.

## 2 Methods

This cross-sectional comparative study evaluated the accuracy, clarity, and completeness of patient-focused educational responses generated by three large language models, namely ChatGPT-4.5 (OpenAI), Gemini Pro 2.5 (Google), and DeepSeek-V3, when answering commonly asked questions related to oral anticoagulant therapy, comparing them against SDERs. The evaluation panel comprised seven experts: two cardiologists with more than 10 years of experience in anticoagulation management and five pharmacists with at least 5 years of experience in a cardiac tertiary hospital. The panel size was consistent with prior studies employing expert evaluation of LLM outputs.^8^, ^9^, ^20^, ^21^, ^22^

### 2.1 Evaluation framework and domain definitions

The evaluation focused on five widely prescribed oral anticoagulants relevant to contemporary cardiovascular practice: warfarin, dabigatran, apixaban, rivaroxaban, and edoxaban. Responses were assessed across three domains considered central to patient education in anticoagulation management: accuracy of medical content, clarity of communication, and completeness of key safety information.^23^ Accuracy of medical content was defined as the degree to which a response contained correct, evidence-based information free from factual errors or clinically misleading statements.^24^ Clarity of communication was defined as the degree to which a response was written in plain, patient-appropriate language, free from unnecessary medical jargon, and logically organized.^23^ Completeness of key safety information was defined as the degree to which a response adequately covered essential safety-relevant information a patient would need to safely use their anticoagulant therapy, including warnings, precautions, and actionable guidance.^23^, ^25^ A mean score ≥ 4 was considered indicative of acceptable response quality across evaluation domains. Responses were independently rated by two blinded expert reviewers using a five-point Likert scale, with detailed scoring descriptors presented in Table 1.

**Table 1:** Five-point Likert scale descriptors for expert evaluation of response quality.

Score	Accuracy of Medical Content	Clarity of Communication	Completeness of Key Safety Information
1	Completely inaccurate; contains major factual errors or clinically misleading statements	Completely unclear; incomprehensible to a patient; heavily laden with medical jargon	Completely incomplete; critical safety information entirely absent
2	Mostly inaccurate; contains several factual errors that may mislead the patient	Mostly unclear; poorly organized and difficult for a patient to understand	Mostly incomplete; major safety information missing
3	Partially accurate; contains minor factual errors but no clinically significant misleading statements	Partially clear; some jargon or organizational issues that may affect patient understanding	Partially complete; some important safety information missing
4	Mostly accurate; minor imprecisions present but no clinically significant errors	Mostly clear; minor language issues that do not impede patient understanding	Mostly complete; only minor omissions of safety information
5	Completely accurate; fully consistent with evidence-based clinical guidance	Completely clear; plain language, well-organized, and easily understood by a patient	Completely complete; all key safety information comprehensively addressed

### 2.2 Identification and selection of FAQs

FAQs related to oral anticoagulant therapy were identified by a clinical pharmacist (Expert 1) from four authoritative sources: the British Heart Foundation, American Heart Association, National Health Service, and Lexicomp.26., 27, 28, 29, 30, 31, 32., 33 Each source was systematically reviewed to extract potential questions addressing key aspects of oral anticoagulant use, including administration, monitoring requirements, adverse effects, drug and food interactions, lifestyle considerations, pregnancy and breastfeeding, comorbid conditions, and periprocedural management.

A preliminary pool of candidate questions was compiled and semantically overlapping items were consolidated. Each candidate question was then cross-checked against the four selected sources, and only questions appearing in at least three of the four sources were retained, as recurrence across multiple authoritative platforms was considered indicative of common patient inquiry and clinical relevance. The complete frequency mapping of candidate questions is provided in the Supplementary file.

Throughout this process, all decisions made by Expert 1 were independently reviewed and confirmed by a cardiologist (Expert 2) to ensure clinical relevance, clarity, and suitability for routine patient counselling, resulting in a final set of 11 FAQs (Table 2).

**Table 2:** Final set of FAQs included for evaluation.

S. no.	Questions
1	How do oral anticoagulants work?
2	How should I take oral anticoagulants?
3	Do I need regular blood tests while taking oral anticoagulants?
4	Will my oral anticoagulant dose need to be adjusted?
5	What will happen if I stop taking oral anticoagulants?
6	What are the potential side effects of oral anticoagulants?
7	Do I need to follow a special diet while taking an oral anticoagulant?
8	Is it safe to take other medications with oral anticoagulants?
9	Can I drink alcohol while taking an oral anticoagulant?
10	Is it safe to take oral anticoagulants during pregnancy or while breastfeeding?
11	Will I need to stop taking oral anticoagulants before the surgery or dental procedures?

### 2.3 Development of SDERs and LLM responses

SDERs were developed by a clinical pharmacist (Expert 3) to reflect evidence-based, patient-appropriate counselling aligned with contemporary cardiovascular practice using authoritative clinical guidelines and reference sources.

SDERs were intended to serve as structured reference responses for patient education rather than as absolute clinical benchmarks, providing a consistent comparison framework grounded in established guidance against which LLM-generated responses could be evaluated.

Following development, the SDERs were independently reviewed and finalized by another cardiologist (Expert 4), who assessed each response for clinical correctness, completeness of patient counselling information, clarity of communication, and safety adequacy. Discrepancies between Expert 3 and Expert 4 were discussed, and responses were revised where necessary to ensure consistency with the same authoritative patient education sources referenced in Section 2.2. The validated SDERs were subsequently used as reference responses for comparative evaluation.

LLM responses were generated by Expert 5 (clinical pharmacist) using ChatGPT-4.5, Gemini Pro 2.5, and DeepSeek-V3 between 1 March and 31 March 2025. All responses were generated through the standard consumer-facing web interfaces of each platform, with no changes made to default model settings such as temperature, top_p, or context window parameters. This was intentional, as it reflects how a typical patient would realistically interact with these tools in everyday practice. To ensure that each response was generated independently, the memory function of each model was disabled before data collection began, a new session was started for each question, and all interactions were carried out in private browsing mode. These steps prevented any influence from previous queries or stored session history on subsequent responses.

A standardized prompt was used across all models and questions to promote consistency in outputs:

*“Provide a patient-friendly answer to the following question at a fifth-grade reading level. Responses should be clear, simple, and medically accurate, avoiding medical jargon. Please keep responses between 50 and 100 words, depending on the complexity of the question. Use a reassuring tone and short sentences to make it easy to understand. Focus on what the patient needs to know and provide actionable advice when needed. Ensure that the information is consistent with trusted medical sources (e.g., NHS, FDA, Lexicomp).”*

### 2.4 Blinding, randomization, and response coding

To minimize evaluation bias, four anonymized annexures were created corresponding to the outputs of ChatGPT-4.5, Gemini Pro 2.5, DeepSeek-V3, and the SDERs. Responses to all FAQs for each anticoagulant were pooled, randomized using a computer-generated sequence, and assigned unique alphanumeric identifiers to conceal their source.

To minimize the risk of bias throughout the study, each expert was assigned a single and distinct role with no overlap across study stages, and no information was shared between experts performing different tasks.

### 2.5 Expert evaluation

Anonymized and randomized responses were independently and blindly evaluated by two clinical pharmacists (Experts 6 and 7). Both experts participated exclusively in the evaluation phase and had no prior involvement in FAQ identification, SDER development, or LLM response generation, ensuring that their assessments were free from any influence related to earlier stages of the study.

Before the formal evaluation began, a calibration exercise was conducted by the principal investigator to ensure that both experts interpreted the rating criteria consistently. Each evaluator was provided with a structured scoring sheet accompanied by illustrative examples for each rating level. They then independently completed practice ratings on a subset of responses, after which a facilitated discussion was held to align their understanding of the criteria before formal assessment commenced.

Following calibration, Experts 6 and 7 independently evaluated all anonymized responses using the three predefined domains and the five-point Likert scale described in Section 2.1 and Table 1. Each response was rated separately for accuracy of medical content, clarity of communication, and completeness of key safety information, without any communication between the two evaluators. Interrater reliability was calculated from these independent ratings. Where differences existed between the two ratings, Experts 6 and 7 subsequently met to discuss and reach a consensus, and the agreed rating was used for all further analyses.

### 2.6 Statistical analysis

Data were recorded in Microsoft Excel and analyzed using SPSS version 29 and RStudio version 4.5.0. Interrater agreement was assessed using linearly weighted Cohen's kappa (κ) to account for ordinal differences in the 1–5 Likert-scale ratings and interpreted according to the criteria proposed by Landis and Koch, where values <0 indicate poor agreement, 0–0.20 slight, 0.21–0.40 fair, 0.41–0.60 moderate, 0.61–0.80 substantial, and 0.81–1.0 almost perfect agreement. Ninety-five percent confidence intervals (CI) were estimated using the standard error of κ, and statistical significance was assessed using the Z test with α = 0.05.

Descriptive statistics are presented as means ± standard deviations. Normality was assessed using the Shapiro–Wilk test, which demonstrated non-normal distribution of the ordinal Likert-scale data (*p* < 0.0001). Accordingly, the Friedman test was used for overall comparisons among the four related response sources, as it is the appropriate non-parametric alternative to repeated-measures ANOVA for paired ordinal data. When the Friedman test was significant, post hoc pairwise comparisons were performed using the Wilcoxon signed-rank test, which is suitable for paired non-parametric data. Because multiple pairwise comparisons increase the likelihood of false-positive findings, *p*-values were adjusted using the Benjamini–Hochberg procedure to control the false discovery rate, defined as the expected proportion of statistically significant results that may represent false positives. Statistical significance was defined as *p* < 0.05.

## 3 Results

### 3.1 Illustrative example of response differences

To illustrate the qualitative differences in responses generated by the three LLMs and the SDER, representative responses to the question *“Can I drink alcohol while taking warfarin?”* are presented in Table 3. This question was selected as it demonstrated clinically meaningful and educationally illustrative differences across response sources in terms of accuracy, clarity, and completeness of patient safety information, and is directly relevant to anticoagulation management given the known interaction between alcohol and warfarin's anticoagulant effect.

**Table 3:** Illustrative comparison of responses to the question “Can I drink alcohol while taking warfarin?” across evaluated response sources.

Source	Response	Accuracy	Clarity	Completeness	Recommended rationale
ChatGPT-4.5	*“You can drink alcohol while taking warfarin, but only in small amounts. Too much alcohol can affect how warfarin works, increasing your risk of bleeding. It's best to limit alcohol to one or two drinks occasionally and avoid heavy or binge drinking. If you drink regularly or want to change your drinking habits, talk to your doctor. They can help you understand what's safe for you. Always take warfarin as prescribed and watch for signs of unusual bleeding, like easy bruising or nosebleeds. If you have any concerns, contact your doctor.”*	4	5	4	Response was medically accurate, well-organized, and easy for patients to understand. It included alcohol limits, bleeding risk, binge drinking warning, and advice to consult a physician. Minor omissions included lack of serving size definitions and explicit INR-related discussion.
DeepSeek V3	*“Drinking alcohol while taking warfarin can be risky. Alcohol can affect how warfarin works and increase your risk of bleeding. If you choose to drink, do so in moderation. For most people, this means no more than 1 drink per day. One drink equals 12 oz of beer, 5 oz of wine, or 1.5 oz of liquor. However, it's best to talk to your doctor first, as they may advise you to avoid alcohol completely based on your health. Always follow their advice to stay safe and keep your warfarin working properly!”*	4	5	4	Response was medically accurate, clearly written, and included practical counselling elements such as serving size definitions and moderation advice. Minor omissions included lack of explicit binge drinking warning and INR-related explanation.
Gemini Pro 2.5	*“It's best to talk to your doctor about drinking alcohol while taking warfarin. Alcohol can sometimes affect how warfarin works and could increase your risk of bleeding. For some people, small amounts of alcohol might be okay, but for others, it might not be safe at all. Your doctor knows your health best and can give you the best advice about drinking alcohol while you are taking warfarin.”*	4	4	3	Response was medically accurate and understandable but comparatively less specific and less actionable. Important counselling details such as alcohol limits, serving size definitions, and binge drinking warnings were not included, reducing overall completeness.
SDER	*“Alcohol in low or moderate amounts (one or two servings per day) is unlikely to have major effects on the INR. People who consume alcohol should limit intake to one to two servings per day. A serving is equal to 1 beer (12 oz), 1 glass of wine (5 oz), or 1.5 oz of spirits. People on warfarin therapy should avoid drinking excessive amounts of alcohol over a short period of time (*e.g.*, with a single meal) because this can affect the INR and increase the risk of injury and serious bleeding.”*	5	5	5	Response was fully consistent with evidence-based guidance and comprehensively addressed key counselling and safety considerations, including INR effects, alcohol limits, serving size definitions, and binge drinking warnings, while remaining clear and patient appropriate.

Ratings represent consensus scores reached through discussion and mutual agreement between two blinded expert reviewers (Expert 6 and Expert 7) using the predefined five-point Likert scale described in Table 1 across three evaluation domains: accuracy of medical content, clarity of communication, and completeness of key safety information.

### 3.2 Interrater reliability assessment

Interrater reliability was assessed using linearly weighted Cohen's kappa (κ) between the two blinded reviewers across all evaluation criteria (accuracy, clarity, and completeness) and response sources (ChatGPT-4.5, DeepSeek-V3, Gemini Pro 2.5, and SDERs) (Table 4). For accuracy, κ values ranged from 0.50 to 0.80. Gemini Pro 2.5 demonstrated the highest agreement (κ = 0.80; 95% CI: 0.60–0.93), followed by SDERs (κ = 0.76; 95% CI: 0.57–0.88) and DeepSeek-V3 (κ = 0.68; 95% CI: 0.48–0.87), all indicating substantial agreement. ChatGPT-4.5 demonstrated moderate agreement (κ = 0.50; 95% CI: 0.22–0.80).

**Table 4:** Linear weighted Cohen's κ for interrater reliability across LLM response sources and evaluation metrics.

Source	Metric	Weighted κ (linear weights)	95% CI	Agreement strength	p value
ChatGPT-4.5	Accuracy	0.50	[0.22, 0.80]	Moderate	<0.001
	Clarity	0.36	[0.16, 0.60]	Fair	<0.001
	Completeness	0.31	[0.12, 0.51]	Fair	<0.001
DeepSeek-V3	Accuracy	0.68	[0.48, 0.87]	Substantial	<0.001
	Clarity	0.50	[0.22, 0.70]	Moderate	<0.001
	Completeness	0.64	[0.41, 0.88]	Substantial	<0.001
Gemini Pro 2.5	Accuracy	0.80	[0.60, 0.93]	Substantial	<0.001
	Clarity	0.82	[0.56, 1.0]	Almost perfect	<0.001
	Completeness	0.74	[0.54, 0.90]	Substantial	<0.001
SDER	Accuracy	0.76	[0.59, 0.88]	Substantial	<0.001
	Clarity	0.50	[0.25, 0.65]	Moderate	<0.001
	Completeness	0.84	[0.64, 1.00]	Almost perfect	<0.001

For clarity, κ values ranged from 0.36 to 0.82. Gemini Pro 2.5 demonstrated almost perfect agreement (κ = 0.82; 95% CI: 0.56–1.00). DeepSeek-V3 (κ = 0.50; 95% CI: 0.22–0.70) and SDERs (κ = 0.50; 95% CI: 0.25–0.65) showed moderate agreement, whereas ChatGPT-4.5 demonstrated fair agreement (κ = 0.36; 95% CI: 0.16–0.60).

For completeness, κ values ranged from 0.31 to 0.84. SDERs demonstrated almost perfect agreement (κ = 0.84; 95% CI: 0.64–1.00), followed by Gemini Pro 2.5 (κ = 0.74; 95% CI: 0.54–0.90) and DeepSeek-V3 (κ = 0.64; 95% CI: 0.41–0.88), both indicating substantial agreement. ChatGPT-4.5 demonstrated fair agreement (κ = 0.31; 95% CI: 0.12–0.51). Overall, agreement was highest for accuracy and completeness and lower for clarity, with ChatGPT-4.5 demonstrating comparatively lower interrater agreement across domains.

ChatGPT-4.5 demonstrated comparatively lower interrater agreement across domains, particularly for clarity and completeness. Descriptive review of rating discrepancies indicated that these differences primarily reflected marginal one-point variations and were more frequently observed for responses addressing nuanced counselling topics such as medication administration guidance, dose adjustment considerations, and side effect profiles, compared with more straightforward factual questions such as routine monitoring requirements.

### 3.3 Overall comparison of response ratings

The Friedman test demonstrated statistically significant differences among the four response sources across all evaluation domains: accuracy (*p* = 0.0022), clarity (*p* = 0.0132), and completeness (*p* < 0.0001) (Table 5).

**Table 5:** Expert ratings across response sources and post hoc pairwise comparisons.

Metric	ChatGPT-4.5 (Mean ± SD)	DeepSeek-V3 (Mean ± SD)	Gemini Pro 2.5 (Mean ± SD)	SDER (Mean ± SD)	Overall *p* value (Friedman)	Comparison group	Post hoc p value (Wilcoxon)	Benjamini-Hochberg adjusted p value
Accuracy	4.76 ± 0.42	4.45 ± 0.68	4.49 ± 0.53	4.61 ± 0.62	**0.0022**	ChatGPT-4.5 > Gemini Pro 2.5	0.002	0.003
						ChatGPT-4.5 > DeepSeek-V3	0.002	0.003
Clarity	4.78 ± 0.41	4.60 ± 0.52	4.52 ± 0.50	4.55 ± 0.56	**0.0132**	ChatGPT-4.5 > Gemini Pro 2.5	<0.01	0.005
						ChatGPT-4.5 > DeepSeek-V3	0.008	0.02
						ChatGPT-4.5 > SDER	0.019	0.02
Completeness	4.85 ± 0.35	4.45 ± 0.68	4.40 ± 0.59	4.61 ± 0.62	**0.000021**	ChatGPT-4.5 > Gemini Pro 2.5	<0.01	0.000089
						ChatGPT-4.5 > DeepSeek-V3	<0.01	0.000855
						ChatGPT-4.5 > SDER	0.01	0.03

Mean ratings are presented as mean ± standard deviation (SD). Only statistically significant pairwise comparisons after Benjamini–Hochberg adjustment are shown.

Across all evaluation domains, ChatGPT-4.5 received the highest mean expert ratings (accuracy: 4.76 ± 0.42; clarity: 4.78 ± 0.41; completeness: 4.85 ± 0.35). Corresponding mean scores were as follows: SDERs (accuracy: 4.61 ± 0.62; clarity: 4.55 ± 0.56; completeness: 4.61 ± 0.62), DeepSeek-V3 (accuracy: 4.45 ± 0.68; clarity: 4.60 ± 0.52; completeness: 4.45 ± 0.68), and Gemini Pro 2.5 (accuracy: 4.49 ± 0.53; clarity: 4.52 ± 0.50; completeness: 4.40 ± 0.59).

Post hoc Wilcoxon signed-rank tests with Benjamini–Hochberg correction identified statistically significant differences in accuracy ratings between ChatGPT-4.5 and Gemini Pro 2.5 (adjusted *p* = 0.003) and between ChatGPT-4.5 and DeepSeek-V3 (adjusted p = 0.003). Statistically significant differences were also observed for clarity between ChatGPT-4.5 and Gemini Pro 2.5 (adjusted *p* = 0.005), DeepSeek-V3 (adjusted *p* = 0.02), and SDERs (adjusted p = 0.02). For completeness, statistically significant differences were identified between ChatGPT-4.5 and Gemini Pro 2.5 (adjusted *p* = 0.000089), DeepSeek-V3 (adjusted *p* = 0.000855), and SDERs (adjusted *p* = 0.03). Only statistically significant pairwise comparisons after Benjamini–Hochberg adjustment are presented in Table 5.

### 3.4 Drug-specific comparison

Drug-specific comparisons of expert ratings across response sources are summarized in Table 6.

**Table 6:** Drug-specific comparison of expert ratings across response sources and post hoc pairwise comparisons.

Drug	Metric	ChatGPT-4.5 (Mean ± SD)	DeepSeek-V3 (Mean ± SD)	Gemini Pro 2.5 (Mean ± SD)	SDER (Mean ± SD)	Overall *p* value (Friedman)	Significant post hoc comparisons (Benjamini-Hochberg adjusted p value)
Warfarin	Accuracy	4.73 ± 0.47	4.27 ± 0.47	3.91 ± 0.30	4.73 ± 0.47	0.001	ChatGPT-4.5 > Gemini Pro 2.5 (0.0201) SDER > Gemini Pro 2.5 (0.025)
	Clarity	4.91 ± 0.30	4.45 ± 0.52	4.45 ± 0.52	4.82 ± 0.40	0.026	–
	Completeness	4.82 ± 0.40	4.27 ± 0.47	3.80 ± 0.42	4.73 ± 0.47	<0.001	ChatGPT-4.5 > Gemini Pro 2.5 (0.01) SDER > Gemini Pro 2.5 (0.01)
Dabigatran	Accuracy	4.82 ± 0.42	4.82 ± 0.42	4.64 ± 0.50	4.45 ± 0.69	0.166	–
	Clarity	4.80 ± 0.40	4.73 ± 0.47	4.45 ± 0.52	4.55 ± 0.52	0.141	–
	Completeness	4.90 ± 0.30	4.82 ± 0.40	4.55 ± 0.52	4.45 ± 0.69	0.104	–
Apixaban	Accuracy	4.55 ± 0.52	4.45 ± 0.82	4.73 ± 0.47	4.64 ± 0.67	0.747	–
	Clarity	4.73 ± 0.47	4.55 ± 0.32	4.55 ± 0.52	4.09 ± 0.54	0.031	–
	Completeness	4.64 ± 0.50	4.45 ± 0.82	4.64 ± 0.50	4.64 ± 0.69	0.838	–
Edoxaban	Accuracy	4.80 ± 0.40	4.36 ± 0.81	4.55 ± 0.52	4.64 ± 0.67	0.179	–
	Clarity	4.73 ± 0.47	4.73 ± 0.47	4.73 ± 0.47	4.64 ± 0.50	0.934	–
	Completeness	4.91 ± 0.30	4.36 ± 0.81	4.45 ± 0.52	4.64 ± 0.67	0.091	–
Rivaroxaban	Accuracy	4.91 ± 0.30	4.36 ± 0.81	4.64 ± 0.52	4.64 ± 0.67	0.169	–
	Clarity	4.73 ± 0.47	4.55 ± 0.69	4.45 ± 0.52	4.64 ± 0.67	0.541	–
	Completeness	5.00 ± 0.00	4.36 ± 0.81	4.55 ± 0.69	4.64 ± 0.67	0.071	–

Mean ratings are presented as mean ± standard deviation (SD). Only statistically significant pairwise comparisons after Benjamini–Hochberg adjustment are presented.

For warfarin, statistically significant overall differences were observed among response sources for accuracy (*p* = 0.001), clarity (*p* = 0.026), and completeness (*p* < 0.001). Post hoc analyses identified significant differences in accuracy between ChatGPT-4.5 and Gemini Pro 2.5 (adjusted *p* = 0.0201) and between SDERs and Gemini Pro 2.5 (adjusted *p* = 0.025). For completeness, statistically significant differences were identified between ChatGPT-4.5 and Gemini Pro 2.5 (adjusted *p* = 0.01) and between SDERs and Gemini Pro 2.5 (adjusted p = 0.01). No statistically significant post hoc differences were identified for clarity after adjustment.

For dabigatran, no statistically significant differences were observed across response sources for accuracy (*p* = 0.166), clarity (*p* = 0.141), or completeness (*p* = 0.104).

For apixaban, a statistically significant overall difference was observed for clarity (*p* = 0.031); however, no pairwise comparisons remained statistically significant following Benjamini–Hochberg adjustment. No statistically significant differences were observed for accuracy (*p* = 0.747) or completeness (*p* = 0.838).

For edoxaban, no statistically significant overall differences were observed across accuracy (*p* = 0.179), clarity (*p* = 0.934), or completeness (*p* = 0.091).

For rivaroxaban, no statistically significant differences were identified across accuracy (*p* = 0.169), clarity (*p* = 0.541), or completeness (*p* = 0.071). Only statistically significant pairwise comparisons after Benjamini–Hochberg adjustment are presented in Table 6.

## 4 Discussion

Interrater reliability analysis revealed varying agreement among expert evaluators across response sources and domains. All Cohen's κ values were statistically significant, indicating that agreement exceeded chance alone.^34^, ^35^ ChatGPT-4.5 demonstrated comparatively lower interrater agreement, particularly for clarity and completeness. Descriptive review of rating discrepancies indicated that these differences primarily reflected marginal one-point variations and were more frequently associated with responses addressing nuanced counselling topics such as medication administration guidance, dose adjustment considerations, and side effect profiles.

This pattern may reflect the greater contextual richness of ChatGPT-generated responses, defined as the ability to address multiple clinically relevant dimensions of a patient query within a single response, including safety considerations, practical counselling, behavioral guidance, and recommendations for professional consultation. For example, in response to the question “Will the dose need to be adjusted?” for warfarin, ChatGPT included guidance regarding INR monitoring, dietary influences, concomitant medications, missed doses, and bleeding precautions within a single response. While both evaluators considered the response high quality and assigned identical accuracy ratings, one evaluator assigned scores of 5 for clarity and completeness whereas the other assigned scores of 4 because additional details such as monitoring frequency and genetic considerations were not explicitly discussed. This illustrates how contextually detailed responses may generate minor yet legitimate variation in subjective assessment domains despite overall agreement regarding factual accuracy.

The comparatively higher agreement observed for accuracy across response sources likely reflects the more objective nature of factual correctness compared with communication-related domains such as clarity and completeness, which inherently involve greater subjective interpretation. Although the evaluators underwent calibration using predefined scoring descriptors, subjective interpretation of broad communication-related constructs may still contribute to minor variability in expert ratings. In addition, ChatGPT-4.5 consistently achieved the highest mean ratings across domains, making small rating differences between scores of 4 and 5 more likely, consistent with potential ceiling effects in high-performing responses. Similar observations have been reported in prior psychometric literature indicating that objective medical accuracy can generally be evaluated more reliably than communication effectiveness.^36^, ^37^

By contrast, DeepSeek-V3 and Gemini Pro 2.5 generally produced more focused and linear responses that addressed fewer dimensions within a single answer, potentially reducing interpretive variability during evaluation and contributing to higher interrater agreement. Similarly, SDERs demonstrated high agreement for completeness but comparatively lower agreement for clarity, likely reflecting their standardized yet more formal structure. Collectively, these findings suggest that interrater agreement may reflect variability in interpretation rather than overall response quality alone, particularly when evaluating nuanced communication-related domains.

Across all sources, ChatGPT received the highest mean expert ratings, with responses generally perceived as more accurate, clear, and comprehensive than those of DeepSeek or Gemini. These results align with prior studies across diverse clinical domains, including osteoporosis,^38^ breast imaging,^39^ hand fracture management,^40^ and endometriosis-related queries,^41^ in which ChatGPT scored better than other models or slightly exceeded standard references. While SDERs demonstrated comparable accuracy, ChatGPT achieved at least equivalent factual reliability. Regarding clarity, ChatGPT consistently demonstrated a relative advantage over Gemini, DeepSeek, and SDERs, reflecting its ability to deliver patient-friendly and comprehensible explanations. Performance may vary by context, as Gemini and DeepSeek have demonstrated higher clarity in pediatric fracture and ACL surgery education.^42^, ^43^ For completeness, ChatGPT was rated more positively than other models and SDERs, reflecting its ability to provide comprehensive patient-oriented information with minimal omission of critical details. This is consistent with studies in dental pulp therapy for immature permanent teeth.^44^ In certain contexts, such as patient education on *Helicobacter pylori*, ChatGPT and SDERs performed equally, indicating that traditional references may match LLM performance under well-defined and standardized knowledge domains.^45^

Analysis of individual oral anticoagulants revealed distinct performance patterns. For warfarin, ChatGPT-4.5 and SDERs achieved higher mean scores than Gemini Pro 2.5 in accuracy and completeness, whereas no significant differences were observed for clarity. This disparity likely reflects warfarin's inherent clinical complexity, arising from its narrow therapeutic index, high interindividual variability influenced by genetics, diet, and comorbidities, and the need for frequent INR monitoring and individualized dose adjustments.^46^, ^47^ Such complexity presents challenges for LLMs, requiring nuanced medical reasoning and integration of multifactorial clinical information. ChatGPT-4.5's stronger performance may reflect its greater capacity to address these complexities while generating clinically relevant and comprehensive explanations.^48^ These findings help explain why performance differences were more apparent for warfarin-related queries than for direct oral anticoagulants.

In contrast, no statistically significant differences were observed among response sources for direct oral anticoagulants. This uniformity may be attributed to the standardized clinical use of DOACs, characterized by fixed dosing regimens, minimal monitoring requirements, and fewer drug interactions. Additionally, DOACs are supported by extensive contemporary randomized clinical trials and clear guideline recommendations, providing more objective, abundant, and recent information readily accessible across LLM platforms.^49^ Overall, these findings suggest that both medication complexity and model capability influence performance differences, whereas more standardized therapies such as DOACs yield comparatively similar outputs across models.

Several factors distinguish our study from prior evaluations. First, previous work often focused on general health information, whereas our study specifically examined oral anticoagulants, a domain requiring nuanced clinical reasoning and safety-focused counselling. Second, we evaluated GPT-4.5, a more recent model incorporating advances in prompt comprehension and reduced hallucination rates. Third, unlike studies relying on simplified scoring systems or single-rater evaluations, we implemented structured multi-rater assessments using predefined evaluation criteria and calibration procedures, providing a more robust and clinically grounded assessment of LLM-generated responses.^50^

All four response sources exceeded the predefined acceptable quality threshold of 4 out of 5 across all three evaluation domains, with mean scores ranging from 4.40 to 4.85. ChatGPT-4.5 consistently achieved the highest mean scores across all domains, followed by SDERs, DeepSeek-V3, and Gemini Pro 2.5. These findings suggest that LLMs may serve as accessible and supplementary sources of patient education information in anticoagulation management, particularly outside routine clinical hours.

However, whether highly rated information quality translates to improved patient understanding, clinical outcomes, or medication adherence warrants further investigation in outcome-based studies. Future research should employ validated patient education assessment tools such as PEMAT or DISCERN to confirm these findings and establish a more robust evidence base for the clinical integration of LLM-generated patient education content.

LLMs should be considered adjuncts rather than replacements for professional medical advice and monitoring, and practical applications may include integration into patient education platforms to provide timely educational support outside routine clinical hours. Future research should focus on standardized protocols for clinical LLM use, safeguards for automated responses, and hybrid systems combining model outputs with clinician oversight. Longitudinal studies assessing patient outcomes will be essential for determining the safety, effectiveness, and acceptability of LLMs in clinical patient education settings.

While this study focused on evaluating the quality of LLM-generated responses, it is equally important to acknowledge the privacy and security risks that arise when patients use publicly accessible LLM platforms to seek health-related guidance. In doing so, patients may unknowingly share sensitive personal information such as their medications, medical conditions, and clinical history with platforms that were not designed to meet healthcare data protection standards. Unlike regulated health information systems, most publicly available LLMs do not offer end-to-end encryption, are not compliant with HIPAA or GDPR, and provide no guarantees against data storage, re-identification, or unauthorized access by third parties.^18^, ^19^ These concerns are especially relevant in anticoagulant therapy, where patients often manage complex and individualized treatment plans and may disclose detailed clinical information when seeking advice.

For LLMs to be safely used alongside clinical systems and electronic health records, they would need to be deployed within secure, privacy-preserving environments. These may include federated learning frameworks, on-premises model hosting, or blockchain-secured data encryption systems that maintain patient data within institution-controlled infrastructure.^19^ Safe integration would also require compatibility with established health data exchange standards, clear mechanisms for clinician oversight, and full compliance with applicable data protection regulations such as HIPAA and GDPR.^18^ Until these conditions are met, the role of LLMs in patient education should remain limited to supervised, non-integrated educational support rather than direct integration into clinical care systems.

## 5 Limitations

This study has several limitations that should be considered when interpreting the findings. First, the analysis focused exclusively on oral anticoagulants, which limits generalizability to other antithrombotic therapies such as injectable agents, including low molecular weight heparins. The results may therefore not be applicable to patient education for other medication classes with different risk profiles or counselling requirements.

Second, although structured evaluation criteria were employed, assessments were based on expert judgment and may not fully capture patient-level comprehension, emotional responses, or real-world interpretive variability. The scoring framework was designed to assess perceived quality of patient-facing information rather than objective patient understanding or behavioral outcomes.

Third, response evaluation was conducted by two blinded expert reviewers. While this approach is commonly used in clinically oriented evaluations of patient education materials, a larger number of evaluators could improve reliability estimates and enhance generalizability. Accordingly, findings should be interpreted as exploratory expert perceptions rather than definitive comparative performance. Future studies incorporating larger multi-expert panels may provide more robust and generalizable assessments.

Fourth, although responses were prompted to approximate a fifth-grade reading level, objective readability metrics were not formally applied. As a result, readability and health literacy alignment were not quantitatively verified and should not be assumed. Future studies may benefit from incorporating standardized readability assessments to further characterize patient accessibility.

Fifth, structural differences between SDERs and LLM-generated outputs may have influenced evaluations of clarity and completeness. Expert responses were designed to prioritize alignment with clinical guidance and safety considerations, whereas LLM outputs were optimized for conversational style and patient-oriented presentation. These inherent differences should be considered when interpreting comparative ratings.

Sixth, patient interactions with LLMs are inherently dynamic and iterative, whereas the present study evaluated responses to static, predefined questions using a standardized prompt. Variations in question phrasing, follow-up queries, or conversational context may influence the accuracy, clarity, and completeness of LLM-generated responses in real-world settings. Accordingly, the structured approach used in this study may overestimate consistency compared with natural patient–LLM interactions.

Finally, not all leading LLM platforms were evaluated, and models such as Claude 3 or LLaMA 3 were not included. In addition, each model generated a single response per question, and reproducibility across multiple response iterations was not assessed. Since responses were generated through standard consumer-facing web interfaces to replicate authentic patient interaction conditions, model-specific parameters such as temperature, top_p, and context window settings were governed by platform defaults and were not user-configurable. These factors should be considered when extrapolating findings to API-based or clinically deployed LLM environments.

## 6 Conclusion

This study achieved its objective of comparatively evaluating the perceived accuracy, clarity, and completeness of LLM-generated responses to common patient questions regarding oral anticoagulant therapy. Overall, LLM-generated responses received high expert ratings across all evaluated sources, with ChatGPT-4.5 achieving the highest overall performance and demonstrating statistically significant advantages across all three evaluation domains. Performance differences were most evident for warfarin-related queries, whereas no statistically significant differences were observed among sources for direct oral anticoagulants. These findings suggest that contemporary LLMs may have potential as supplementary tools for patient education in anticoagulation management, particularly for structured and commonly encountered patient queries. However, the findings were obtained under controlled evaluation conditions using static FAQs and may not fully reflect the complexity of real-world patient interactions, which are often dynamic and individualized. Privacy, security, regulatory compliance, and appropriate clinician oversight remain important considerations before these tools can be responsibly integrated into clinical settings. Further research incorporating validated patient education assessment tools, real-world conversational settings, and patient outcome measures will be important to confirm the clinical value and practical applicability of LLM-supported patient education.

## Clinical trial number

Not applicable.

## CRediT authorship contribution statement

**Mohammed Amer Khan:** Writing – review & editing, Writing – original draft, Visualization, Validation, Software, Methodology, Investigation, Formal analysis, Data curation, Conceptualization. **Vaibhav Chaudhary:** Writing – review & editing, Methodology, Investigation. **Mohammed Maazuddin:** Investigation, Formal analysis, Data curation. **Ihtisham Sultan:** Visualization, Validation, Software, Investigation. **Saamiya Mehnaaz:** Writing – original draft, Investigation, Data curation. **Biplab Pal:** Writing – review & editing, Validation, Supervision, Project administration, Conceptualization.

## Consent for publication

All authors give consent for publication.

## Ethics approval and consent to participate

Ethics committee approval or exemption was not required for this study, as it did not involve human participants, identifiable personal or medical data, or any direct interaction with individuals, and was conducted entirely using fully anonymized data, consistent with the ICMR National Ethical Guidelines for Biomedical and Health Research Involving Human Participants (2017) and the principles of the Declaration of Helsinki.

## Funding

The authors received no specific funding for this work.

## Declaration of competing interest

The authors declare that they have no known competing financial interests or personal relationships that could have appeared to influence the work reported in this paper.

## Data Availability

All relevant data are contained within the manuscript and in its supplementary materials.
