# Supplementary material for: Evaluation of large language model responses to patient questions on oral anticoagulant therapy: a comparative expert assessment
Source: Explor Res Clin Soc Pharm. 2026 Jun 9;23:100812. doi: 10.1016/j.rcsop.2026.100812 (PMC13279031; doi:10.1016/j.rcsop.2026.100812)
Supplement: Supplementary file 1 — Supplementary material [file mmc1.docx]

Table 1: Supplementary Table 1. Frequency mapping of candidate frequently asked questions (FAQs) across authoritative sources

| S.no | Questions | Lexicomp | British Heart Foundation | National health sciences | American Heart Association |
| --- | --- | --- | --- | --- | --- |
| 1 | How do oral anticoagulants work? |  |  |  |  |
| 2 | What are the types of oral anticoagulant? |  |  |  |  |
| 3 | How should I take oral anticoagulants? |  |  |  |  |
| 4 | Do I need regular blood tests while taking oral anticoagulants? |  |  |  |  |
| 5 | Will my oral anticoagulant dose need to be adjusted? |  |  |  |  |
| 6 | What will happen if I stop taking oral anticoagulants? |  |  |  |  |
| 7 | What are the potential side effects of oral anticoagulants? |  |  |  |  |
| 8 | Is there a medicine to reverse the effects if I have serious bleeding? |  |  |  |  |
| 9 | Do I need to follow a special diet while taking an oral anticoagulant? |  |  |  |  |
| 10 | What if I have kidney or liver disease? |  |  |  |  |
| 11 | Is it safe to take other medications with oral anticoagulants? |  |  |  |  |
| 12 | Can I drink alcohol while taking an oral anticoagulant? |  |  |  |  |
| 13 | Is it safe to take oral anticoagulants during pregnancy or while breastfeeding? |  |  |  |  |
| 14 | Will I need to stop taking oral anticoagulants before the surgery or dental procedures? |  |  |  |  |

**ANNEXURE -A: ChatGPT Reponses**

**Warfarin**

**How does warfarin work?**

Warfarin is a medicine that helps prevent blood clots. It works by slowing down your body's ability to form clots. Your blood needs vitamin K to make clots, and warfarin blocks vitamin K from working as usual. This helps keep your blood flowing smoothly and lowers the risk of serious problems like strokes or heart attacks. Because warfarin affects clotting, you may need regular blood tests to make sure you're taking the right amount. Always take warfarin exactly as your doctor tells you, and talk to them before making any changes to your diet or other medicines.

**How should I take it?**

Take warfarin exactly as your doctor tells you. Usually, you take it once a day at the same time, with or without food. Your dose may change based on blood tests, so follow your doctor’s instructions carefully. Never skip a dose or take extra to make up for a missed one. Some foods and medicines can affect warfarin, so ask your doctor before making any changes. If you forget a dose, take it as soon as you remember on the same day. If it's the next day, skip the missed dose and tell your doctor.

**Do I need regular blood tests?**

Yes, you need regular blood tests while taking warfarin. These tests check how well your blood is clotting and help your doctor adjust your dose if needed. Keeping your blood at the right level is important to prevent bleeding or blood clots. At first, you may need tests often, but over time, they may be less frequent. Always go to your scheduled tests and follow your doctor’s advice. If you notice unusual bruising, bleeding, or feel unwell, contact your doctor right away.

**Will the dose need to be adjusted?**

Yes, your warfarin dose may need to be adjusted. Everyone’s body responds differently, so your doctor will check your blood regularly to make sure you’re taking the right amount. Your dose may change if your diet, health, or other medicines affect how warfarin works. It’s important to take it exactly as prescribed and not change your dose on your own. If you miss a dose or notice unusual bleeding or bruising, tell your doctor. Regular check-ups help keep you safe and ensure warfarin is working properly.

**What will happen if I stop taking it?**

If you stop taking warfarin without your doctor’s advice, your risk of blood clots may go up. This could lead to serious problems like a stroke, heart attack, or blood clot in your lungs or legs. If your doctor decides you no longer need warfarin, they will guide you on how to stop safely. Never stop on your own, even if you feel fine. If you’re worried about side effects or have trouble taking warfarin, talk to your doctor. They can help you find the safest option for your health.

**What are the potential side effects?**

Warfarin can cause side effects, but not everyone gets them. The most common one is bleeding, which can show as easy bruising, nosebleeds, bleeding gums, or longer bleeding from cuts. Serious bleeding, like blood in your urine, stool, or vomit, needs medical help right away. Other rare side effects include skin rashes or hair loss. Tell your doctor if you have signs of bleeding or feel unwell. Regular blood tests help keep your dose safe. If you have any concerns, talk to your doctor—they can help adjust your dose or find solutions.

**Do I need to do anything special about my diet while taking it?**

Yes, while taking warfarin, you should keep your diet steady, especially foods with vitamin K, like green leafy vegetables (spinach, kale, broccoli). Vitamin K can affect how warfarin works, so sudden changes in how much you eat can make warfarin less effective or increase bleeding risk. You don’t have to avoid these foods—just eat them consistently. Also, avoid large amounts of alcohol and talk to your doctor before taking supplements or herbal products. If you have questions about your diet, your doctor or a dietitian can help you make safe choices.

**is it safe to take other medications?**

Some medications can affect how warfarin works, so always check with your doctor or pharmacist before taking anything new. This includes prescription drugs, over-the-counter medicines, vitamins, and herbal supplements. Some medicines can increase your risk of bleeding, while others can make warfarin less effective. Pain relievers like ibuprofen and aspirin can be risky unless your doctor says they’re okay. Always let your doctor know about any medications you take, even common cold medicines or supplements. If you notice unusual bleeding or bruising, contact your doctor right away.

**Can I drink alcohol while taking it? If yes, how much is safe to take?**

You can drink alcohol while taking warfarin, but only in small amounts. Too much alcohol can affect how warfarin works, increasing your risk of bleeding. It’s best to limit alcohol to one or two drinks occasionally and avoid heavy or binge drinking. If you drink regularly or want to change your drinking habits, talk to your doctor. They can help you understand what’s safe for you. Always take warfarin as prescribed and watch for signs of unusual bleeding, like easy bruising or nosebleeds. If you have any concerns, contact your doctor.

**What if I am pregnant or breast feeding?**

Warfarin is not safe during pregnancy because it can harm the baby, so if you are pregnant or planning to be, tell your doctor right away—they may switch you to a safer medicine. However, warfarin is usually safe while breastfeeding since only a small amount passes into breast milk, but you should still check with your doctor to make sure it's the best option for you and your baby.

**Will I need to stop the medicine before surgery or dental procedures?**

You may need to stop taking warfarin before surgery or certain dental procedures to lower the risk of bleeding, but this depends on the type of procedure and your health condition. Always tell your doctor or dentist that you take warfarin before any planned procedure, and they will let you know if you need to stop or adjust your dose. Never stop warfarin on your own, as this could increase your risk of blood clots. Your doctor will guide you on when to stop and restart it safely.

**Apixaban**

**How does Apixaban work?**

Apixaban is a blood thinner that helps prevent clots from forming in your body. It works by blocking a protein called Factor Xa, which is needed for blood to clot. This lowers the risk of dangerous clots that can cause strokes or block blood flow to the lungs or legs. Unlike some other blood thinners, apixaban does not require regular blood tests. Always take it exactly as your doctor tells you, and do not stop taking it without medical advice, as this could increase your risk of blood clots.

**How should I take it?**

Take apixaban exactly as your doctor tells you. It is usually taken once or twice a day, with or without food. Try to take it at the same time each day to help you remember. Swallow the tablet whole with water—do not crush or chew it unless your doctor says it’s okay. If you miss a dose, take it as soon as you remember on the same day, but do not take two doses at once. Never stop taking apixaban without talking to your doctor, as this could increase your risk of blood clots.

**Do I need regular blood tests?**

No, you do not need regular blood tests while taking apixaban. Unlike warfarin, apixaban does not require routine monitoring to check blood clotting levels. However, your doctor may occasionally check your kidney and liver function to make sure the medicine is safe for you. Always take apixaban as prescribed and let your doctor know if you experience unusual bleeding, bruising, or any other concerns.

**Will the dose need to be adjusted?**

Your apixaban dose may need to be adjusted based on your age, weight, kidney function, or other medications you take. Your doctor will decide the right dose for you and may change it if your health condition changes. Never adjust your dose on your own, and always follow your doctor’s instructions to keep the medicine working safely. If you have any concerns, talk to your doctor.

**What will happen if I stop taking it?**

If you stop taking apixaban without your doctor’s advice, your risk of blood clots may increase, which could lead to serious problems like a stroke or a clot in your lungs or legs. If your doctor decides you should stop taking it, they will guide you on how to do so safely. Never stop on your own, even if you feel fine. If you have concerns or side effects, talk to your doctor about the best options for you.

**What are the potential side effects?**

The most common side effect of apixaban is bleeding, which may cause easy bruising, nosebleeds, bleeding gums, or longer bleeding from cuts. Serious bleeding, such as blood in your urine, stool, or vomit, needs immediate medical attention. Some people may also experience nausea or feel more tired than usual. Rarely, allergic reactions can occur, with symptoms like swelling or trouble breathing—seek help right away if this happens. If you notice unusual bleeding or feel unwell, contact your doctor as soon as possible.

**Do I need to do anything special about my diet while taking it?**

No, you don’t need to make any special changes to your diet while taking apixaban. Unlike warfarin, apixaban is not affected by vitamin K, so you can eat your usual foods. However, it’s best to drink alcohol in moderation and check with your doctor before taking supplements or herbal products, as some may affect how apixaban works. If you have any concerns about your diet, talk to your doctor or pharmacist.

**is it safe to take other medications?**

Some medications can affect how apixaban works, so always check with your doctor or pharmacist before starting or stopping any medicine. This includes prescription drugs, over-the-counter medicines, vitamins, and herbal supplements. Certain pain relievers (like ibuprofen and aspirin) and other blood thinners can increase your risk of bleeding. Some medicines may also make apixaban less effective. Always let your doctor know about any medications you take, and if you notice unusual bleeding or bruising, contact your doctor right away.

**Can I drink alcohol while taking it? If yes, how much is safe to take?**

You can drink alcohol while taking apixaban, but it’s best to do so in moderation. Too much alcohol can increase your risk of bleeding and make bruising worse. If you choose to drink, limit it to one or two drinks occasionally and avoid heavy or binge drinking. If you drink regularly or have concerns, talk to your doctor to make sure it’s safe for you.

**What if I am pregnant or breast feeding?**

Apixaban is not recommended during pregnancy because it may harm the baby, so if you are pregnant or planning to become pregnant, talk to your doctor about safer options. It is also unknown if apixaban passes into breast milk, so breastfeeding while taking it is not usually advised. Always check with your doctor to find the safest choice for you and your baby.

**Will I need to stop the medicine before surgery or dental procedures?**

You may need to stop taking apixaban before surgery or certain dental procedures to reduce the risk of bleeding, but this depends on the type of procedure and your health condition. Always tell your doctor or dentist that you take apixaban before any planned procedure, and they will let you know if and when you should stop or restart it. Never stop taking apixaban on your own, as this could increase your risk of blood clots.

**Dabigatran**

**How does Dabigatran work?**

Dabigatran is a blood thinner that helps prevent clots from forming in your body. It works by blocking a protein called thrombin, which is needed for blood to clot. This helps lower the risk of serious problems like strokes or blood clots in the legs or lungs. Unlike some other blood thinners, dabigatran does not require regular blood tests. Always take it exactly as your doctor tells you, and do not stop taking it without medical advice, as this could increase your risk of dangerous clots.

**How should I take it?**

Take dabigatran exactly as your doctor tells you. Swallow the capsule whole with a full glass of water—do not crush, chew, or open it, as this can affect how the medicine works. Usually, you take it once or twice a day, with or without food. Try to take it at the same time each day to help you remember. If you miss a dose, take it as soon as you remember unless it’s almost time for your next dose—never take two doses at once. Always follow your doctor’s advice and ask them if you have any questions.

**Do I need regular blood tests?**

No, you do not need regular blood tests while taking dabigatran like you would with warfarin. However, your doctor may check your kidney function and overall health from time to time to make sure the medicine is working safely for you. Always take dabigatran as prescribed and let your doctor know if you have any unusual bleeding, bruising, or other concerns.

**Will the dose need to be adjusted?**

Your dabigatran dose may need to be adjusted based on your kidney function, age, weight, or other medicines you take. Your doctor will choose the right dose for you and may change it if your health condition changes. Never adjust your dose on your own, and always follow your doctor’s instructions to keep the medicine working safely. If you have any concerns, talk to your doctor.

**What will happen if I stop taking it?**

If you stop taking dabigatran without your doctor’s advice, your risk of blood clots may increase, which could lead to serious problems like a stroke or a clot in your lungs or legs. If your doctor decides you should stop taking it, they will guide you on how to do so safely. Never stop on your own, even if you feel fine. If you have concerns or side effects, talk to your doctor about the best options for you.

**What are the potential side effects?**

The most common side effect of dabigatran is bleeding, which can show as easy bruising, nosebleeds, bleeding gums, or longer bleeding from cuts. Serious bleeding, such as blood in your urine, stool, or vomit, needs immediate medical attention. Some people may also have stomach pain, indigestion, or nausea. Rarely, dabigatran can cause an allergic reaction with symptoms like swelling or trouble breathing—get help right away if this happens. If you notice any unusual bleeding or feel unwell, contact your doctor as soon as possible.

**Do I need to do anything special about my diet while taking it?**

No, you don’t need to make any special changes to your diet while taking dabigatran. Unlike warfarin, dabigatran is not affected by vitamin K, so you can eat your usual foods. However, it’s best to take it with a full glass of water and avoid alcohol or excessive caffeine if they upset your stomach. Always talk to your doctor before taking supplements or herbal products, as some may affect how dabigatran works. If you have any questions about your diet, your doctor or pharmacist can help.

**is it safe to take other medications?**

Some medications can affect how dabigatran works, so always check with your doctor or pharmacist before starting or stopping any medicine. This includes prescription drugs, over-the-counter medicines, vitamins, and herbal supplements. Some medicines, like certain pain relievers (e.g., ibuprofen, aspirin) and blood thinners, can increase your risk of bleeding. Others may make dabigatran less effective. Always let your doctor know about any medications you take, and if you notice unusual bleeding or bruising, contact your doctor right away.

**Can I drink alcohol while taking it? If yes, how much is safe to take?**

You can drink alcohol while taking dabigatran, but it’s best to do so in moderation. Too much alcohol can increase your risk of bleeding and affect how well the medicine works. If you choose to drink, limit it to one or two drinks occasionally and avoid heavy or binge drinking. If you have any concerns or drink alcohol regularly, talk to your doctor to make sure it’s safe for you.

**What if I am pregnant or breast feeding?**

Dabigatran is not recommended during pregnancy because it may harm the baby, so if you are pregnant or planning to become pregnant, talk to your doctor about safer options. It is also not known if dabigatran passes into breast milk, so breastfeeding while taking it is not usually advised. Always check with your doctor to find the safest choice for you and your baby.

**Will I need to stop the medicine before surgery or dental procedures?**

You may need to stop taking dabigatran before surgery or certain dental procedures to reduce the risk of bleeding, but this depends on the type of procedure and your health condition. Always tell your doctor or dentist that you take dabigatran before any planned procedure, and they will let you know if and when you should stop or restart it. Never stop taking dabigatran on your own, as this could increase your risk of blood clots.

**Rivaroxaban**

**How does rivaroxaban work?**

Rivaroxaban is a blood thinner that helps prevent clots from forming in your body. It works by blocking a protein called Factor Xa, which is needed for blood to clot. This lowers the risk of serious problems like strokes or blood clots in the legs or lungs. Unlike some other blood thinners, rivaroxaban does not require regular blood tests. Always take it exactly as your doctor tells you, and do not stop taking it without medical advice, as this could increase your risk of blood clots.

**How should I take it?**

Take rivaroxaban exactly as your doctor tells you. It is usually taken once or twice a day, depending on your condition. If your dose is 15 mg or more, take it with food to help your body absorb it properly. Swallow the tablet whole with water—do not crush or chew it unless your doctor says it’s okay. Try to take it at the same time each day to help you remember. If you miss a dose, follow your doctor’s instructions. Never stop taking rivaroxaban without talking to your doctor, as this could increase your risk of blood clots.

**Do I need regular blood tests?**

No, you do not need regular blood tests while taking rivaroxaban. Unlike warfarin, rivaroxaban does not require routine monitoring to check blood clotting levels. However, your doctor may occasionally check your kidney and liver function to ensure the medicine is safe and working well for you. Always take rivaroxaban as prescribed and let your doctor know if you experience unusual bleeding, bruising, or other concerns.

**Will the dose need to be adjusted?**

Your rivaroxaban dose may need to be adjusted based on your kidney function, age, weight, or other medications you take. Your doctor will decide the right dose for you and may change it if your health condition changes. Never adjust your dose on your own, and always follow your doctor’s instructions to keep the medicine working safely. If you have any concerns, talk to your doctor.

**What will happen if I stop taking it?**

If you stop taking rivaroxaban without your doctor’s advice, your risk of blood clots may increase, which could lead to serious problems like a stroke or a clot in your lungs or legs. If your doctor decides you should stop taking it, they will guide you on how to do so safely. Never stop on your own, even if you feel fine. If you have concerns or side effects, talk to your doctor about the best options for you.

**What are the potential side effects?**

The most common side effect of rivaroxaban is bleeding, which may cause easy bruising, nosebleeds, bleeding gums, or longer bleeding from cuts. Serious bleeding, such as blood in your urine, stool, or vomit, needs immediate medical attention. Some people may also experience nausea, dizziness, or feel more tired than usual. Rarely, allergic reactions can occur, with symptoms like swelling or trouble breathing—seek help right away if this happens. If you notice unusual bleeding or feel unwell, contact your doctor as soon as possible.

**Do I need to do anything special about my diet while taking it?**

No, you don’t need to make any special changes to your diet while taking rivaroxaban. Unlike warfarin, rivaroxaban is not affected by vitamin K, so you can eat your usual foods. However, if your dose is 15 mg or more, you must take it with food to help your body absorb it properly. It’s also best to drink alcohol in moderation and check with your doctor before taking supplements or herbal products, as some may affect how rivaroxaban works. If you have any concerns about your diet, talk to your doctor or pharmacist.

**is it safe to take other medications?**

Some medications can affect how rivaroxaban works, so always check with your doctor or pharmacist before starting or stopping any medicine. This includes prescription drugs, over-the-counter medicines, vitamins, and herbal supplements. Certain pain relievers (like ibuprofen and aspirin) and other blood thinners can increase your risk of bleeding. Some medicines may also make rivaroxaban less effective. Always let your doctor know about any medications you take, and if you notice unusual bleeding or bruising, contact your doctor right away.

**Can I drink alcohol while taking it? If yes, how much is safe to take?**

You can drink alcohol while taking rivaroxaban, but it’s best to do so in moderation. Too much alcohol can increase your risk of bleeding and make bruising worse. If you choose to drink, limit it to one or two drinks occasionally and avoid heavy or binge drinking. If you drink regularly or have concerns, talk to your doctor to make sure it’s safe for you.

**What if I am pregnant or breast feeding?**

Rivaroxaban is not recommended during pregnancy because it may harm the baby, so if you are pregnant or planning to become pregnant, talk to your doctor about safer options. It is also unknown if rivaroxaban passes into breast milk, so breastfeeding while taking it is not usually advised. Always check with your doctor to find the safest choice for you and your baby.

**Will I need to stop the medicine before surgery or dental procedures?**

You may need to stop taking rivaroxaban before surgery or certain dental procedures to lower the risk of bleeding, but this depends on the type of procedure and your health condition. Always tell your doctor or dentist that you take rivaroxaban before any planned procedure, and they will let you know if and when you should stop or restart it. Never stop taking rivaroxaban on your own, as this could increase your risk of blood clots.

**Edoxaban**

**How does edoxaban work?**

Edoxaban is a blood thinner that helps prevent clots from forming in your body. It works by blocking a protein called Factor Xa, which is needed for blood to clot. This lowers the risk of serious problems like strokes or blood clots in the legs or lungs. Unlike some other blood thinners, edoxaban does not require regular blood tests. Always take it exactly as your doctor tells you, and do not stop taking it without medical advice, as this could increase your risk of blood clots.

**How should I take it?**

Take edoxaban exactly as your doctor tells you. It is usually taken once a day, with or without food. Swallow the tablet whole with water—do not crush or chew it unless your doctor says it’s okay. Try to take it at the same time each day to help you remember. If you miss a dose, take it as soon as you remember on the same day, but do not take two doses at once. Never stop taking edoxaban without talking to your doctor, as this could increase your risk of blood clots.

**Do I need regular blood tests?**

No, you do not need regular blood tests while taking edoxaban. Unlike warfarin, edoxaban does not require routine monitoring to check blood clotting levels. However, your doctor may occasionally check your kidney function and overall health to ensure the medicine is safe and working well for you. Always take edoxaban as prescribed and let your doctor know if you experience unusual bleeding, bruising, or other concerns.

**Will the dose need to be adjusted?**

Your edoxaban dose may need to be adjusted based on your kidney function, weight, age, or other medications you take. Your doctor will decide the right dose for you and may change it if your health condition changes. Never adjust your dose on your own, and always follow your doctor’s instructions to keep the medicine working safely. If you have any concerns, talk to your doctor.

**What will happen if I stop taking it?**

If you stop taking edoxaban without your doctor’s advice, your risk of blood clots may increase, which could lead to serious problems like a stroke or a clot in your lungs or legs. If your doctor decides you should stop taking it, they will guide you on how to do so safely. Never stop on your own, even if you feel fine. If you have concerns or side effects, talk to your doctor about the best options for you.

**What are the potential side effects?**

The most common side effect of edoxaban is bleeding, which may cause easy bruising, nosebleeds, bleeding gums, or longer bleeding from cuts. Serious bleeding, such as blood in your urine, stool, or vomit, needs immediate medical attention. Some people may also experience nausea or feel more tired than usual. Rarely, allergic reactions can occur, with symptoms like swelling or trouble breathing—seek help right away if this happens. If you notice unusual bleeding or feel unwell, contact your doctor as soon as possible.

**Do I need to do anything special about my diet while taking it?**

No, you don’t need to make any special changes to your diet while taking edoxaban. Unlike warfarin, edoxaban is not affected by vitamin K, so you can eat your usual foods. However, it’s best to drink alcohol in moderation and check with your doctor before taking supplements or herbal products, as some may affect how edoxaban works. If you have any concerns about your diet, talk to your doctor or pharmacist.

**is it safe to take other medications?**

Some medications can affect how edoxaban works, so always check with your doctor or pharmacist before starting or stopping any medicine. This includes prescription drugs, over-the-counter medicines, vitamins, and herbal supplements. Certain pain relievers (like ibuprofen and aspirin) and other blood thinners can increase your risk of bleeding. Some medicines may also make edoxaban less effective. Always let your doctor know about any medications you take, and if you notice unusual bleeding or bruising, contact your doctor right away.

**Can I drink alcohol while taking it? If yes, how much is safe to take?**

You can drink alcohol while taking edoxaban, but it’s best to do so in moderation. Too much alcohol can increase your risk of bleeding and make bruising worse. If you choose to drink, limit it to one or two drinks occasionally and avoid heavy or binge drinking. If you drink regularly or have concerns, talk to your doctor to make sure it’s safe for you.

**What if I am pregnant or breast feeding?**

Edoxaban is not recommended during pregnancy because it may harm the baby, so if you are pregnant or planning to become pregnant, talk to your doctor about safer options. It is also unknown if edoxaban passes into breast milk, so breastfeeding while taking it is not usually advised. Always check with your doctor to find the safest choice for you and your baby.

**Will I need to stop the medicine before surgery or dental procedures?**

You may need to stop taking edoxaban before surgery or certain dental procedures to lower the risk of bleeding, but this depends on the type of procedure and your health condition. Always tell your doctor or dentist that you take edoxaban before any planned procedure, and they will let you know if and when you should stop or restart it. Never stop taking edoxaban on your own, as this could increase your risk of blood clots.

**ANNEXURE -B: Deep seek Reponses**

**Warfarin**

**How does warfarin work?**

Warfarin is a medicine that helps stop blood clots from forming in your body. It works by slowing down your body’s ability to make clotting factors, which are like tiny helpers that make your blood stick together. This helps keep your blood flowing smoothly. You’ll need regular blood tests to make sure the dose is just right. Always take it as your doctor says and avoid big changes in your diet, like eating lots of leafy greens, unless your doctor says it’s okay.

**How should I take it?**

Take warfarin exactly as your doctor tells you. Usually, it’s once a day, at the same time each day. You can take it with or without food. Don’t skip doses or change the amount without asking your doctor. Keep all your appointments for blood tests to check how well it’s working. Tell your doctor about any other medicines, vitamins, or herbs you’re taking, as they can affect warfarin. If you miss a dose, call your doctor for advice. Always follow their instructions carefully!

**Do I need regular blood tests?**

Yes, you’ll need regular blood tests called INR tests while taking warfarin. These tests check how well your blood is clotting and help your doctor make sure your dose is just right. At first, you might need tests often, like once a week, but once your levels are stable, they may be less frequent. It’s important to keep up with these tests to stay safe and make sure warfarin is working properly. Always follow your doctor’s advice about when to get tested!

**Will the dose need to be adjusted?**

Yes, your warfarin dose might need to be adjusted sometimes. This is normal! The dose depends on your INR blood test results, which show how well your blood is clotting. If your INR is too high or too low, your doctor may change your dose to keep it in the right range. Things like diet, other medicines, or illness can also affect your dose. Always take the exact dose your doctor prescribes and let them know if anything changes, like new medicines or health issues. They’ll help keep your dose just right!

**What will happen if I stop taking it?**

If you stop taking warfarin without your doctor’s advice, you could be at risk of forming dangerous blood clots. These clots can cause serious problems, like a stroke or heart attack. Never stop taking warfarin or change your dose on your own. If you’re having side effects or want to stop, talk to your doctor first. They might switch you to a different medicine or help you stop safely. Always follow your doctor’s instructions to stay healthy and avoid risks!

**What are the potential side effects?**

Warfarin can cause some side effects. The most common is bleeding, like nosebleeds, bleeding gums, or bruising more easily. Serious bleeding, like coughing up blood or dark stools, needs immediate medical attention. Rarely, warfarin can cause skin rash or hair loss. To stay safe, avoid activities that could cause injury, like contact sports, and use a soft toothbrush. Tell your doctor if you notice unusual bleeding or other side effects. They can help manage these and make sure warfarin is working well for you. Always follow their advice!

**Do I need to do anything special about my diet while taking it?**

Yes, you should be careful with your diet while taking warfarin. Foods high in vitamin K, like leafy greens (spinach, kale, broccoli), can affect how warfarin works. You don’t need to avoid these foods completely, but try to eat the same amount consistently. Sudden changes can mess with your INR levels. Also, limit alcohol and avoid cranberry juice, as they can interfere with warfarin. Talk to your doctor or a dietitian if you’re unsure about what to eat. They can help you plan a balanced diet that keeps you healthy and your warfarin working well!

**Is it safe to take other medications?**

Not all medicines are safe to take with warfarin. Some can make warfarin work too well or not well enough, which can be dangerous. Always tell your doctor or pharmacist about any other medicines, vitamins, or herbs you’re taking, even over-the-counter ones like pain relievers or cold medicine. They’ll check if it’s safe to take them with warfarin. Never start or stop any medicine without asking your doctor first. They’ll help you avoid problems and keep you safe while taking warfarin!

**Can I drink alcohol while taking it? If yes, how much is safe to take?**

Drinking alcohol while taking warfarin can be risky. Alcohol can affect how warfarin works and increase your risk of bleeding. If you choose to drink, do so in moderation. For most people, this means no more than 1 drink per day. One drink equals 12 ounces of beer, 5 ounces of wine, or 1.5 ounces of liquor. However, it’s best to talk to your doctor first, as they may advise you to avoid alcohol completely based on your health. Always follow their advice to stay safe and keep your warfarin working properly!

**What if I am pregnant or breast feeding?**

If you’re pregnant or planning to become pregnant, tell your doctor right away. Warfarin can harm an unborn baby, especially in the first trimester and near delivery. Your doctor may switch you to a safer medicine, like heparin, during pregnancy. If you’re breastfeeding, warfarin is usually considered safe because only tiny amounts pass into breast milk. However, always check with your doctor first to make sure it’s okay for you and your baby. They’ll help you find the best and safest option for your situation!

**Will I need to stop the medicine before surgery or dental procedures?**

Yes, you’ll likely need to stop warfarin before surgery or dental procedures to reduce the risk of bleeding. However, never stop taking it without your doctor’s instructions. They’ll tell you when to stop and when to start again after the procedure. In some cases, they may switch you to a different medicine temporarily. Always tell your surgeon or dentist that you’re taking warfarin so they can plan accordingly. Your doctor will make sure you stay safe and your blood clots properly during and after the procedure!

**Apixaban**

**How does Apixaban work?**

Apixaban is a medicine that helps prevent blood clots. It works by blocking a substance in your blood called Factor Xa, which helps your blood clot. By slowing down Factor Xa, apixaban keeps your blood from clotting too much. This lowers your risk of dangerous clots that can cause strokes or other problems. Unlike warfarin, apixaban doesn’t require regular blood tests, but you still need to take it exactly as your doctor says. Always tell your doctor about other medicines you’re taking, as some can affect how apixaban works.

**How should I take it?**

Take apixaban exactly as your doctor tells you. Usually, it’s taken twice a day, with or without food. Swallow the tablet whole with water—don’t crush, chew, or split it. Try to take it at the same times each day to keep the medicine working well. If you miss a dose, take it as soon as you remember, but skip it if it’s almost time for your next dose. Never take extra to make up for a missed one. Always follow your doctor’s instructions and let them know if you have any side effects or concerns!

**Do I need regular blood tests?**

No, you don’t need regular blood tests with apixaban like you do with warfarin. Apixaban works in a more predictable way, so your doctor won’t need to check your blood clotting levels as often. However, your doctor might still order occasional tests to check your kidneys, since apixaban is cleared from your body through them. Always take apixaban exactly as prescribed and let your doctor know if you have any unusual symptoms, like bleeding or bruising. They’ll make sure the medicine is working safely for you!

**Will the dose need to be adjusted?**

Yes, your apixaban dose might need to be adjusted based on your age, weight, kidney function, or other health conditions. Your doctor will decide the right dose for you when you start the medicine. If your health changes, like if your kidneys aren’t working as well, your doctor may adjust your dose. Always take the exact dose your doctor prescribes and don’t change it on your own. Let your doctor know if you’re taking other medicines or have any new health issues, as these can affect how apixaban works. They’ll keep your dose just right for you!

**What will happen if I stop taking it?**

If you stop taking apixaban without your doctor’s advice, you could be at risk of forming dangerous blood clots. These clots can cause serious problems, like a stroke or deep vein thrombosis (DVT). Never stop taking apixaban or change your dose on your own. If you’re having side effects or want to stop, talk to your doctor first. They might switch you to a different medicine or help you stop safely. Always follow your doctor’s instructions to stay healthy and avoid risks!

**What are the potential side effects?**

Apixaban can cause some side effects. The most common is bleeding, like nosebleeds, bleeding gums, or bruising more easily. Serious bleeding, such as coughing up blood, dark stools, or heavy periods, needs immediate medical attention. Other possible side effects include nausea, anaemia, or rash. Rarely, it can cause an allergic reaction. To stay safe, avoid activities that could cause injury, like contact sports, and use a soft toothbrush. Tell your doctor if you notice unusual bleeding or other side effects. They can help manage these and make sure apixaban is working well for you!

**Do I need to do anything special about my diet while taking it?**

No, you don’t need to make special diet changes while taking apixaban, unlike with warfarin. You can eat normally, including foods high in vitamin K, like leafy greens. However, it’s still important to maintain a healthy, balanced diet. Avoid excessive alcohol, as it can increase your risk of bleeding. If you have questions about your diet or are unsure about certain foods, talk to your doctor or a dietitian. They can help you plan meals that keep you healthy while taking apixaban. Always follow their advice to stay safe!

**Is it safe to take other medications?**

Not all medicines are safe to take with apixaban. Some can increase your risk of bleeding or affect how apixaban works. Always tell your doctor or pharmacist about any other medicines, vitamins, or herbs you’re taking, even over-the-counter ones like pain relievers or cold medicine. They’ll check if it’s safe to take them with apixaban. Never start or stop any medicine without asking your doctor first. They’ll help you avoid problems and keep you safe while taking apixaban!

**Can I drink alcohol while taking it? If yes, how much is safe to take?**

Drinking alcohol while taking apixaban can increase your risk of bleeding. If you choose to drink, do so in moderation. For most people, this means no more than 1 drink per day. One drink equals 12 ounces of beer, 5 ounces of wine, or 1.5 ounces of liquor. However, it’s best to talk to your doctor first, as they may advise you to avoid alcohol completely based on your health. Always follow their advice to stay safe and keep your apixaban working properly!

**What if I am pregnant or breast feeding?**

If you’re pregnant or planning to become pregnant, tell your doctor right away. Apixaban is not recommended during pregnancy because its effects on an unborn baby are not fully known. Your doctor may switch you to a safer medicine, like heparin, during pregnancy. If you’re breastfeeding, it’s not clear if apixaban passes into breast milk, so talk to your doctor before taking it. They’ll help you find the best and safest option for you and your baby. Always follow their advice to protect your health and your baby’s!

**Will I need to stop the medicine before surgery or dental procedures?**

Yes, you’ll likely need to stop apixaban before surgery or dental procedures to reduce the risk of bleeding. However, never stop taking it without your doctor’s instructions. They’ll tell you when to stop and when to start again after the procedure. The timing depends on your kidney function and the type of procedure. Always tell your surgeon or dentist that you’re taking apixaban so they can plan accordingly. Your doctor will make sure you stay safe and your blood clots properly during and after the procedure!

**Dabigatran**

**How does dabigatran work?**

Dabigatran is a medicine that helps prevent blood clots. It works by blocking a substance in your blood called thrombin, which helps your blood clot. By slowing down thrombin, dabigatran keeps your blood from clotting too much. This lowers your risk of dangerous clots that can cause strokes or other problems. Unlike warfarin, dabigatran doesn’t require regular blood tests, but you still need to take it exactly as your doctor says. Always tell your doctor about other medicines you’re taking, as some can affect how dabigatran works.

**How should I take it?**

Take dabigatran exactly as your doctor tells you. Usually, it’s taken twice a day, with or without food. Swallow the capsule whole with water—don’t crush, chew, or open it. Try to take it at the same times each day to keep the medicine working well. If you miss a dose, take it as soon as you remember, but skip it if it’s almost time for your next dose. Never take extra to make up for a missed one. Always follow your doctor’s instructions and let them know if you have any side effects or concerns!

**Do I need regular blood tests?**

No, you don’t need regular blood tests with dabigatran like you do with warfarin. Dabigatran works in a more predictable way, so your doctor won’t need to check your blood clotting levels as often. However, your doctor might still order occasional tests to check your kidneys, since dabigatran is cleared from your body through them. Always take dabigatran exactly as prescribed and let your doctor know if you have any unusual symptoms, like bleeding or bruising. They’ll make sure the medicine is working safely for you!

**Will the dose need to be adjusted?**

Yes, your dabigatran dose might need to be adjusted based on your age, kidney function, or other health conditions. Your doctor will decide the right dose for you when you start the medicine. If your health changes, like if your kidneys aren’t working as well, your doctor may adjust your dose. Always take the exact dose your doctor prescribes and don’t change it on your own. Let your doctor know if you’re taking other medicines or have any new health issues, as these can affect how dabigatran works. They’ll keep your dose just right for you!

**What will happen if I stop taking it?**

If you stop taking dabigatran without your doctor’s advice, you could be at risk of forming dangerous blood clots. These clots can cause serious problems, like a stroke or deep vein thrombosis (DVT). Never stop taking dabigatran or change your dose on your own. If you’re having side effects or want to stop, talk to your doctor first. They might switch you to a different medicine or help you stop safely. Always follow your doctor’s instructions to stay healthy and avoid risks!

**What are the potential side effects?**

Dabigatran can cause some side effects. The most common is bleeding, like nosebleeds, bleeding gums, or bruising more easily. Serious bleeding, such as coughing up blood, dark stools, or heavy periods, needs immediate medical attention. Other possible side effects include stomach upset, indigestion, or nausea. Rarely, it can cause an allergic reaction. To stay safe, avoid activities that could cause injury, like contact sports, and use a soft toothbrush. Tell your doctor if you notice unusual bleeding or other side effects. They can help manage these and make sure dabigatran is working well for you!

**Do I need to do anything special about my diet while taking it?**

No, you don’t need to make special diet changes while taking dabigatran, unlike with warfarin. You can eat normally, including foods high in vitamin K, like leafy greens. However, it’s still important to maintain a healthy, balanced diet. Avoid excessive alcohol, as it can increase your risk of bleeding. If you have questions about your diet or are unsure about certain foods, talk to your doctor or a dietitian. They can help you plan meals that keep you healthy while taking dabigatran. Always follow their advice to stay safe!

**Is it safe to take other medications?**

Not all medicines are safe to take with dabigatran. Some can increase your risk of bleeding or affect how dabigatran works. Always tell your doctor or pharmacist about any other medicines, vitamins, or herbs you’re taking, even over-the-counter ones like pain relievers or cold medicine. They’ll check if it’s safe to take them with dabigatran. Never start or stop any medicine without asking your doctor first. They’ll help you avoid problems and keep you safe while taking dabigatran!

**Can I drink alcohol while taking it? If yes, how much is safe to take?**

Drinking alcohol while taking dabigatran can increase your risk of bleeding. If you choose to drink, do so in moderation. For most people, this means no more than 1 drink per day. One drink equals 12 ounces of beer, 5 ounces of wine, or 1.5 ounces of liquor. However, it’s best to talk to your doctor first, as they may advise you to avoid alcohol completely based on your health. Always follow their advice to stay safe and keep your dabigatran working properly!

**What if I am pregnant or breast feeding?**

If you’re pregnant or planning to become pregnant, tell your doctor right away. Dabigatran is not recommended during pregnancy because its effects on an unborn baby are not fully known. Your doctor may switch you to a safer medicine, like heparin, during pregnancy. If you’re breastfeeding, it’s not clear if dabigatran passes into breast milk, so talk to your doctor before taking it. They’ll help you find the best and safest option for you and your baby. Always follow their advice to protect your health and your baby’s!

**Will I need to stop the medicine before surgery or dental procedures?**

Yes, you’ll likely need to stop dabigatran before surgery or dental procedures to reduce the risk of bleeding. However, never stop taking it without your doctor’s instructions. They’ll tell you when to stop and when to start again after the procedure. The timing depends on your kidney function and the type of procedure. Always tell your surgeon or dentist that you’re taking dabigatran so they can plan accordingly. Your doctor will make sure you stay safe and your blood clots properly during and after the procedure!

**Rivaroxaban**

**How does rivaroxaban work?**

Rivaroxaban is a medicine that helps prevent blood clots. It works by blocking a substance in your blood called Factor Xa, which helps your blood clot. By slowing down Factor Xa, rivaroxaban keeps your blood from clotting too much. This lowers your risk of dangerous clots that can cause strokes or other problems. Unlike warfarin, rivaroxaban doesn’t require regular blood tests, but you still need to take it exactly as your doctor says. Always tell your doctor about other medicines you’re taking, as some can affect how rivaroxaban works.

**How should I take it?**

Take rivaroxaban exactly as your doctor tells you. For most conditions, it’s taken once a day with food to help your body absorb it better. Swallow the tablet whole with water—don’t crush, chew, or split it. Try to take it at the same time each day to keep the medicine working well. If you miss a dose, take it as soon as you remember, but skip it if it’s almost time for your next dose. Never take extra to make up for a missed one. Always follow your doctor’s instructions and let them know if you have any side effects or concerns!

**Do I need regular blood tests?**

No, you don’t need regular blood tests with rivaroxaban like you do with warfarin. Rivaroxaban works in a more predictable way, so your doctor won’t need to check your blood clotting levels as often. However, your doctor might still order occasional tests to check your kidneys, since rivaroxaban is cleared from your body through them. Always take rivaroxaban exactly as prescribed and let your doctor know if you have any unusual symptoms, like bleeding or bruising. They’ll make sure the medicine is working safely for you!

**Will the dose need to be adjusted?**

Yes, your rivaroxaban dose might need to be adjusted based on your age, weight, kidney function, or other health conditions. Your doctor will decide the right dose for you when you start the medicine. If your health changes, like if your kidneys aren’t working as well, your doctor may adjust your dose. Always take the exact dose your doctor prescribes and don’t change it on your own. Let your doctor know if you’re taking other medicines or have any new health issues, as these can affect how rivaroxaban works. They’ll keep your dose just right for you!

**What will happen if I stop taking it?**

If you stop taking rivaroxaban without your doctor’s advice, you could be at risk of forming dangerous blood clots. These clots can cause serious problems, like a stroke or deep vein thrombosis (DVT). Never stop taking rivaroxaban or change your dose on your own. If you’re having side effects or want to stop, talk to your doctor first. They might switch you to a different medicine or help you stop safely. Always follow your doctor’s instructions to stay healthy and avoid risks!

**What are the potential side effects?**

Rivaroxaban can cause some side effects. The most common is bleeding, like nosebleeds, bleeding gums, or bruising more easily. Serious bleeding, such as coughing up blood, dark stools, or heavy periods, needs immediate medical attention. Other possible side effects include stomach pain, nausea, or rash. Rarely, it can cause an allergic reaction. To stay safe, avoid activities that could cause injury, like contact sports, and use a soft toothbrush. Tell your doctor if you notice unusual bleeding or other side effects. They can help manage these and make sure rivaroxaban is working well for you!

**Do I need to do anything special about my diet while taking it?**

No, you don’t need to make special diet changes while taking rivaroxaban, unlike with warfarin. You can eat normally, including foods high in vitamin K, like leafy greens. However, it’s still important to maintain a healthy, balanced diet. Avoid excessive alcohol, as it can increase your risk of bleeding. If you have questions about your diet or are unsure about certain foods, talk to your doctor or a dietitian. They can help you plan meals that keep you healthy while taking rivaroxaban. Always follow their advice to stay safe!

**Is it safe to take other medications?**

Not all medicines are safe to take with rivaroxaban. Some can increase your risk of bleeding or affect how rivaroxaban works. Always tell your doctor or pharmacist about any other medicines, vitamins, or herbs you’re taking, even over-the-counter ones like pain relievers or cold medicine. They’ll check if it’s safe to take them with rivaroxaban. Never start or stop any medicine without asking your doctor first. They’ll help you avoid problems and keep you safe while taking rivaroxaban!

**Can I drink alcohol while taking it? If yes, how much is safe to take?**

Drinking alcohol while taking rivaroxaban can increase your risk of bleeding. If you choose to drink, do so in moderation. For most people, this means no more than 1 drink per day. One drink equals 12 ounces of beer, 5 ounces of wine, or 1.5 ounces of liquor. However, it’s best to talk to your doctor first, as they may advise you to avoid alcohol completely based on your health. Always follow their advice to stay safe and keep your rivaroxaban working properly!

**What if I am pregnant or breast feeding?**

If you’re pregnant or planning to become pregnant, tell your doctor right away. Rivaroxaban is not recommended during pregnancy because its effects on an unborn baby are not fully known. Your doctor may switch you to a safer medicine, like heparin, during pregnancy. If you’re breastfeeding, it’s not clear if rivaroxaban passes into breast milk, so talk to your doctor before taking it. They’ll help you find the best and safest option for you and your baby. Always follow their advice to protect your health and your baby’s!

**Will I need to stop the medicine before surgery or dental procedures?**

Yes, you’ll likely need to stop rivaroxaban before surgery or dental procedures to reduce the risk of bleeding. However, never stop taking it without your doctor’s instructions. They’ll tell you when to stop and when to start again after the procedure. The timing depends on your kidney function and the type of procedure. Always tell your surgeon or dentist that you’re taking rivaroxaban so they can plan accordingly. Your doctor will make sure you stay safe and your blood clots properly during and after the procedure!

**Edoxaban**

**How does edoxaban work?**

Edoxaban is a medicine that helps prevent blood clots. It works by blocking a substance in your blood called Factor Xa, which helps your blood clot. By slowing down Factor Xa, edoxaban keeps your blood from clotting too much. This lowers your risk of dangerous clots that can cause strokes or other problems. Unlike warfarin, edoxaban doesn’t require regular blood tests, but you still need to take it exactly as your doctor says. Always tell your doctor about other medicines you’re taking, as some can affect how edoxaban works.

**How should I take it?**

Take edoxaban exactly as your doctor tells you. Usually, it’s taken once a day, with or without food. Swallow the tablet whole with water—don’t crush, chew, or split it. Try to take it at the same time each day to keep the medicine working well. If you miss a dose, take it as soon as you remember, but skip it if it’s almost time for your next dose. Never take extra to make up for a missed one. Always follow your doctor’s instructions and let them know if you have any side effects or concerns!

**Do I need regular blood tests?**

No, you don’t need regular blood tests with edoxaban like you do with warfarin. Edoxaban works in a more predictable way, so your doctor won’t need to check your blood clotting levels as often. However, your doctor might still order occasional tests to check your kidneys, since edoxaban is cleared from your body through them. Always take edoxaban exactly as prescribed and let your doctor know if you have any unusual symptoms, like bleeding or bruising. They’ll make sure the medicine is working safely for you!

**Will the dose need to be adjusted?**

Yes, your edoxaban dose might need to be adjusted based on your age, weight, kidney function, or other health conditions. Your doctor will decide the right dose for you when you start the medicine. If your health changes, like if your kidneys aren’t working as well, your doctor may adjust your dose. Always take the exact dose your doctor prescribes and don’t change it on your own. Let your doctor know if you’re taking other medicines or have any new health issues, as these can affect how edoxaban works. They’ll keep your dose just right for you!

**What will happen if I stop taking it?**

If you stop taking edoxaban without your doctor’s advice, you could be at risk of forming dangerous blood clots. These clots can cause serious problems, like a stroke or deep vein thrombosis (DVT). Never stop taking edoxaban or change your dose on your own. If you’re having side effects or want to stop, talk to your doctor first. They might switch you to a different medicine or help you stop safely. Always follow your doctor’s instructions to stay healthy and avoid risks!

**What are the potential side effects?**

Edoxaban can cause some side effects. The most common is bleeding, like nosebleeds, bleeding gums, or bruising more easily. Serious bleeding, such as coughing up blood, dark stools, or heavy periods, needs immediate medical attention. Other possible side effects include nausea, anaemia, or rash. Rarely, it can cause an allergic reaction. To stay safe, avoid activities that could cause injury, like contact sports, and use a soft toothbrush. Tell your doctor if you notice unusual bleeding or other side effects. They can help manage these and make sure edoxaban is working well for you!

**Do I need to do anything special about my diet while taking it?**

No, you don’t need to make special diet changes while taking edoxaban, unlike with warfarin. You can eat normally, including foods high in vitamin K, like leafy greens. However, it’s still important to maintain a healthy, balanced diet. Avoid excessive alcohol, as it can increase your risk of bleeding. If you have questions about your diet or are unsure about certain foods, talk to your doctor or a dietitian. They can help you plan meals that keep you healthy while taking edoxaban. Always follow their advice to stay safe!

**Is it safe to take other medications?**

Not all medicines are safe to take with edoxaban. Some can increase your risk of bleeding or affect how edoxaban works. Always tell your doctor or pharmacist about any other medicines, vitamins, or herbs you’re taking, even over-the-counter ones like pain relievers or cold medicine. They’ll check if it’s safe to take them with edoxaban. Never start or stop any medicine without asking your doctor first. They’ll help you avoid problems and keep you safe while taking edoxaban!

**Can I drink alcohol while taking it? If yes, how much is safe to take?**

Drinking alcohol while taking edoxaban can increase your risk of bleeding. If you choose to drink, do so in moderation. For most people, this means no more than 1 drink per day. One drink equals 12 ounces of beer, 5 ounces of wine, or 1.5 ounces of liquor. However, it’s best to talk to your doctor first, as they may advise you to avoid alcohol completely based on your health. Always follow their advice to stay safe and keep your edoxaban working properly!

**What if I am pregnant or breast feeding?**

If you’re pregnant or planning to become pregnant, tell your doctor right away. Edoxaban is not recommended during pregnancy because its effects on an unborn baby are not fully known. Your doctor may switch you to a safer medicine, like heparin, during pregnancy. If you’re breastfeeding, it’s not clear if edoxaban passes into breast milk, so talk to your doctor before taking it. They’ll help you find the best and safest option for you and your baby. Always follow their advice to protect your health and your baby’s!

**Will I need to stop the medicine before surgery or dental procedures?**

Yes, you’ll likely need to stop edoxaban before surgery or dental procedures to reduce the risk of bleeding. However, never stop taking it without your doctor’s instructions. They’ll tell you when to stop and when to start again after the procedure. The timing depends on your kidney function and the type of procedure. Always tell your surgeon or dentist that you’re taking edoxaban so they can plan accordingly. Your doctor will make sure you stay safe and your blood clots properly during and after the procedure!

**ANNEXURE -C: Gemini Reponses**

**Warfarin**

**How does warfarin work?**

Warfarin is medicine to help keep your blood flowing smoothly. Think of it as helping to thin your blood a little. Your body uses vitamin K to make clots, which can sometimes be too much. Warfarin blocks vitamin K, so your body makes fewer clots. This helps stop bad clots from forming and keeps your blood moving the way it should. Be sure to take it exactly as your doctor says and get your blood checked regularly.

**How should I take it?**

Take your warfarin exactly how your doctor tells you to. Try to take it at the same time each day, to help you remember. You can take it with or without food. If you miss a dose, call your doctor or pharmacist right away to ask what to do. Don't stop taking it unless your doctor says it's okay, even if you feel fine. It's very important to get your blood checked regularly, so the doctor knows if your dose is right. If you have questions, just ask your doctor or pharmacist!

**Do I need regular blood tests?**

Yes, you will need regular blood tests while you are taking warfarin. These tests are very important! They help your doctor see how well the warfarin is working to thin your blood. The tests tell your doctor if your dose of warfarin is just right, too high, or too low. If your dose is too high, you could bleed too easily. If it's too low, you could still get dangerous blood clots. So, keep all your appointments for blood tests, so your doctor can keep you safe and healthy!

**Will the dose need to be adjusted?**

Yes, your warfarin dose might need to be changed from time to time. Because the blood tests tell your doctor how well the medicine is working, they might need to adjust your dose to make sure your blood is just right. Lots of things can affect how warfarin works, like what you eat, other medicines you take, and even how your body changes. Don't worry if your dose changes, it just means your doctor is paying close attention to keep you safe and healthy! Always follow your doctor's instructions carefully.

**What will happen if I stop taking it?**

If you stop taking warfarin without talking to your doctor first, your blood could start to clot too much again. This could be dangerous, because it could lead to problems like a stroke or a blood clot in your leg or lungs. Warfarin is helping to prevent those bad clots from forming. So, it's very important to talk to your doctor before you stop taking warfarin. They will tell you if it's safe to stop, and they might need to change your other medicines or check you more closely.

**What are the potential side effects?**

Warfarin can sometimes cause side effects, but it's important to remember that not everyone gets them. The most common side effect is bleeding more easily. This could mean nosebleeds, bleeding gums, or bruises that show up easily.

If you notice any of these, tell your doctor. Other, less common side effects can also happen. It's best to talk to your doctor or pharmacist about all the possible side effects, so you know what to watch out for. They can answer your questions and help you manage any problems that might come up.

**Do I need to do anything special about my diet while taking it?**

Yes, it's good to keep your diet fairly steady while taking warfarin, especially with foods that have vitamin K. Vitamin K is in leafy green vegetables like spinach, kale, and broccoli. Warfarin works by blocking vitamin K, so big changes in how much vitamin K you eat can affect how well the medicine works. You don't have to avoid these foods, just try to eat about the same amount each week. Talk to your doctor or a dietitian for more advice about your diet while on warfarin. They can help you make a plan that works for you!

**Is it safe to take other medications?**

That's a very important question! It's really important to tell your doctor about all the other medicines you take, including vitamins and supplements. Some medicines can change how warfarin works, either making it too strong or not strong enough. Even common medicines like aspirin or ibuprofen can cause problems. Your doctor will check to make sure it's safe to take all your medicines together. Never start or stop a medicine without talking to your doctor first! They want to keep you safe and healthy.

**Can I drink alcohol while taking it? If yes, how much is safe to take?**

It's best to talk to your doctor about drinking alcohol while taking warfarin. Alcohol can sometimes affect how warfarin works and could increase your risk of bleeding. For some people, small amounts of alcohol might be okay, but for others, it might not be safe at all. Your doctor knows your health best and can give you the best advice about drinking alcohol while you are taking warfarin.

**What if I am pregnant or breast feeding?**

It's very important to talk to your doctor right away if you are pregnant, planning to become pregnant, or breastfeeding while taking warfarin. Warfarin can be harmful to a baby during pregnancy, so your doctor may need to change your medicine. It's usually not recommended to take warfarin while breastfeeding either. Your doctor will help you decide on the safest plan for you and your baby. They will make sure you get the right treatment to protect both of your health.

**Will I need to stop the medicine before surgery or dental procedures?**

Yes, you might need to stop taking warfarin before a surgery or dental procedure. It depends on the type of procedure and your risk of bleeding. Your doctor will tell you exactly what to do. They might have you stop taking warfarin a few days before and then restart it afterward. Sometimes, they might switch you to another medicine for a short time. Always tell your surgeon or dentist that you are taking warfarin, so they can talk to your doctor and make a safe plan for you.

**Apixaban**

**How does apixaban work?**

Apixaban is a medicine that helps keep your blood from clotting too much. It's like a helper that thins your blood a little. Your blood has special helpers that make clots, like tiny builders. Apixaban blocks one of those helpers, called Factor Xa. By blocking Factor Xa, apixaban makes it harder for clots to form. This helps prevent dangerous clots. Take it exactly as your doctor tells you, and ask them or your pharmacist if you have questions.

**How should I take it?**

Take apixaban exactly as your doctor tells you, usually twice a day about 12 hours apart, with or without food. If you have trouble swallowing, you can crush the tablet and mix it with water, juice, or applesauce and take it right away. If you miss a dose, take it when you remember unless it's almost time for your next one, but don't double up. Keep taking it even if you feel well unless your doctor says to stop. If you have any questions, just ask your doctor or pharmacist.

**Do I need regular blood tests?**

Usually, you **don't need regular blood tests** while taking apixaban, like you do with warfarin. This is a good thing for many people! However, your doctor might still want to check your blood sometimes, especially if you have kidney problems or if they are worried about bleeding. So, keep going to your appointments, so your doctor can make sure the medicine is working safely for you.

**Will the dose need to be adjusted?**

Yes, your apixaban dose might need to be changed by your doctor. This often happens if you have kidney problems, because your kidneys help remove the medicine. Also, if you are taking other medicines that can change how apixaban works, your doctor might adjust your dose. It's important to tell your doctor about all the medicines you take, so they can make sure your apixaban dose is right for you.

**What will happen if I stop taking it?**

If you stop taking apixaban without talking to your doctor first, your blood could start to clot too much again. Apixaban helps prevent dangerous clots, so stopping it could put you at risk for a stroke or other serious problems. It's important to talk to your doctor before you stop taking apixaban, so they can tell you if it's safe and help you find another way to prevent blood clots if needed. Always talk to your doctor first.

**What are the potential side effects?**

Apixaban can sometimes cause side effects, but many people don't have any serious problems. The most common side effect is bleeding more easily. This could mean nosebleeds, bruising more easily, or bleeding gums when you brush your teeth.

If you notice any unusual bleeding or other new problems, tell your doctor right away. They can help you manage any side effects and make sure you are safe. Remember, the good things about taking apixaban usually outweigh the risks of side effects.

**Do I need to do anything special about my diet while taking it?**

Great news! You usually don't need to make big changes to your diet while taking apixaban. It's not affected by vitamin K in foods like leafy green vegetables, like warfarin is. Just try to eat a healthy, balanced diet like you normally would.

If you have any questions about your diet and apixaban, talk to your doctor or pharmacist. They can give you advice that's just right for you.

**Is it safe to take other medications?**

It's super important to tell your doctor about all the other medicines you take, even over-the-counter ones like pain relievers, and also vitamins and supplements. Some medicines can change how apixaban works in your body, making it too strong or not strong enough. Your doctor needs to know everything you're taking to keep you safe and make sure your medicines work well together. Never start or stop a medicine without talking to your doctor first!

**Can I drink alcohol while taking it? If yes, how much is safe to take?**

It's a good idea to talk to your doctor about drinking alcohol while you're taking apixaban. Alcohol can sometimes increase your risk of bleeding, and apixaban also makes you bleed more easily. So, drinking alcohol while taking apixaban might increase that risk. Your doctor knows your health best and can tell you if it's safe to drink alcohol and how much is okay for you.

**What if I am pregnant or breast feeding?**

It's very important to talk to your doctor right away if you are pregnant, planning to get pregnant, or breastfeeding while taking apixaban. Apixaban might not be safe for your baby during pregnancy or while breastfeeding. Your doctor will help you decide on the safest plan for you and your baby. They might need to change your medicine or find another way to prevent blood clots during this time. Your doctor's goal is to keep both you and your baby healthy!

**Will I need to stop the medicine before surgery or dental procedures?**

Yes, you will likely need to stop taking apixaban before surgery or certain dental procedures. Because apixaban makes it easier to bleed, stopping it for a short time helps lower the risk of bleeding during the procedure. Your doctor will tell you exactly when to stop and when to restart apixaban. Make sure your surgeon or dentist knows you're taking apixaban, so they can talk to your doctor and plan things safely.

**Dabigatran**

**How does dabigatran work?**

Dabigatran is a medicine to help keep your blood from clotting too much. It helps thin your blood by blocking a special part called "thrombin." Thrombin helps make clots, but dabigatran stops it from working. This makes it harder for clots to form and helps prevent dangerous ones. Take it exactly as your doctor tells you, and ask them or your pharmacist if you have questions.

**How should I take it?**

Take dabigatran exactly as your doctor says, usually twice a day about 12 hours apart. Swallow the capsules whole, don't chew or open them, and you can take it with or without food. If you miss a dose, take it when you remember unless it's almost time for your next one, but don't double up. Keep taking it even if you feel well, unless your doctor tells you to stop, and always store it in its original container. Ask your doctor or pharmacist if you have any questions.

**Do I need regular blood tests?**

Usually, you **do not need regular blood tests** while taking dabigatran, like you do with warfarin. This is one of the good things about it! However, your doctor might still want to check your blood sometimes, especially if you have kidney problems or if they are worried about bleeding. So, it's important to go to all your appointments, so your doctor can make sure the medicine is working safely for you.

**Will the dose need to be adjusted?**

Yes, your doctor might need to change your dose of dabigatran sometimes. This usually happens if you have problems with your kidneys, because your kidneys help get the medicine out of your body. If your kidneys aren't working as well, you might need a lower dose. Also, your doctor might change your dose if you're taking other medicines that can affect how dabigatran works. It's important to tell your doctor about all the medicines you take, so they can make sure your dose of dabigatran is right for you.

**What will happen if I stop taking it?**

If you stop taking dabigatran without talking to your doctor first, your blood could start to clot too much again. Dabigatran helps prevent dangerous clots, so stopping it could put you at risk for a stroke or other serious problems. It's important to talk to your doctor before you stop taking dabigatran, so they can tell you if it's safe and help you find another way to prevent blood clots if needed. Don't stop taking it on your own!

**What are the potential side effects?**

Dabigatran can have some side effects, but most people don't have serious problems. The most common side effect is bleeding more easily. This could be things like nosebleeds, bruising, or bleeding gums when you brush your teeth.

Some people might also have stomach upset or indigestion. If you notice any unusual bleeding or other new problems, tell your doctor right away. They can help you manage any side effects and make sure you are safe. It's important to remember that the benefits of taking dabigatran usually outweigh the risks of side effects.

If you notice any of these, tell your doctor. Other, less common side effects can also happen. It's best to talk to your doctor or pharmacist about all the possible side effects, so you know what to watch out for. They can answer your questions and help you manage any problems that might come up.

**Do I need to do anything special about my diet while taking it?**

Good news! You don't usually need to make big changes to your diet while taking dabigatran. Unlike warfarin, dabigatran isn't affected by vitamin K in foods like leafy green vegetables. Just eat a healthy, balanced diet like usual. However, it's always a good idea to talk to your doctor or pharmacist if you have any questions about your diet and dabigatran. They can give you personalized advice based on your health and other medicines you take.

**Is it safe to take other medications?**

It's super important to tell your doctor about all the other medicines you take, even over-the-counter ones like pain relievers, and also any vitamins or supplements. Some medicines can change how dabigatran works in your body, making it too strong or not strong enough. Your doctor needs to know everything you're taking so they can make sure it's safe for you and that your medicines work well together. Never start or stop a medicine without talking to your doctor first!

**Can I drink alcohol while taking it? If yes, how much is safe to take?**

It's best to talk to your doctor about drinking alcohol while you're taking dabigatran. Alcohol can sometimes increase your risk of bleeding, and dabigatran also makes you bleed more easily. So, drinking alcohol while taking dabigatran could increase that risk even more. Your doctor knows your health best and can tell you if it's safe to drink alcohol and how much is okay for you.

**What if I am pregnant or breast feeding?**

It's very important to talk to your doctor right away if you are pregnant, planning to get pregnant, or breastfeeding while taking dabigatran. Dabigatran might not be safe for your baby during pregnancy or while breastfeeding. Your doctor will help you decide on the safest plan for you and your baby. They might need to change your medicine or find another way to prevent blood clots during this time. Your doctor's goal is to keep both you and your baby healthy!

**Will I need to stop the medicine before surgery or dental procedures?**

Yes, you will likely need to stop taking dabigatran before surgery or certain dental procedures. Because dabigatran makes it easier to bleed, stopping it for a short time helps lower the risk of bleeding during the procedure. Your doctor will tell you exactly when to stop and when to restart dabigatran. Make sure your surgeon or dentist knows you're taking dabigatran, so they can talk to your doctor and plan things safely.

**Rivaroxaban**

**How does rivaroxaban work?**

Rivaroxaban is a medicine to help keep your blood from clotting too much. Think of it as a helper that thins your blood a little. Your blood has special parts that help make clots, like tiny builders. Rivaroxaban blocks one of those parts, called Factor Xa. By blocking Factor Xa, rivaroxaban makes it harder for clots to form. This helps prevent dangerous clots from happening. Take it exactly as your doctor tells you, and ask your doctor or pharmacist if you have any questions.

**How should I take it?**

Take rivaroxaban exactly as your doctor says; if it's the 15mg or 20mg tablet, take it once a day with your evening meal. If it's the 2.5mg or 10mg, you can take it with or without food. Swallow the tablet whole, don't crush it, and if you have trouble swallowing, talk to your doctor. If you miss a dose, take it the same day, but don't double up. Keep taking it even if you feel well, unless your doctor tells you to stop. Ask your doctor or pharmacist if you have any questions.

**Do I need regular blood tests?**

Usually, you **don't need regular blood tests** while taking rivaroxaban, like people taking warfarin do. This is often seen as a plus! but your doctor might still want to check your blood sometimes, especially if you have kidney problems or if they are worried about bleeding. It's important to keep going to your checkups, so your doctor can make sure the medicine is working safely for you.

**Will the dose need to be adjusted?**

Yes, your doctor might need to change your dose of rivaroxaban sometimes. This could happen if you have kidney problems, because your kidneys help get the medicine out of your body. Also, certain other medicines can change how rivaroxaban works, so your doctor might need to adjust your dose if you're taking those too. It's important to tell your doctor about all the medicines you take, so they can make sure your rivaroxaban dose is just right.

**What will happen if I stop taking it?**

If you stop taking rivaroxaban without talking to your doctor first, your blood could start to clot too much again. Rivaroxaban helps prevent dangerous blood clots, so stopping it could put you at risk for problems like a stroke. It's important to talk to your doctor before you stop taking rivaroxaban, so they can tell you if it's safe and help you find another way to prevent blood clots if needed. Always talk to your doctor before stopping.

**What are the potential side effects?**

Rivaroxaban can sometimes cause side effects, but most people don't have serious problems. The most common side effect is bleeding more easily. This might mean nosebleeds, bruising more easily, or bleeding gums when you brush your teeth.

If you notice any unusual bleeding or other new problems, tell your doctor right away. They can help you manage any side effects and make sure you're safe. Remember, the good things about taking rivaroxaban usually outweigh the risks of side effects

**Do I need to do anything special about my diet while taking it?**

Good news! You usually don't need to do anything special with your diet while taking rivaroxaban. It's not affected by vitamin K in foods like leafy green vegetables. So just try to eat a healthy, balanced diet like you normally would. If you have any questions about your diet and rivaroxaban, talk to your doctor or pharmacist. They can give you advice that's just right for you.

**Is it safe to take other medications?**

It's really important to tell your doctor about all the other medicines you take, even over-the-counter ones like pain relievers, and also vitamins and supplements. Some medicines can change how rivaroxaban works in your body, making it too strong or not strong enough. Your doctor needs to know everything you're taking to keep you safe and make sure your medicines work well together. Never start or stop a medicine without talking to your doctor first!

**Can I drink alcohol while taking it? If yes, how much is safe to take?**

It's a good idea to talk to your doctor about drinking alcohol while you're taking rivaroxaban. Alcohol can sometimes increase your risk of bleeding, and rivaroxaban also makes you bleed more easily. So, drinking alcohol while taking rivaroxaban might make that risk bigger. Your doctor knows your health best and can tell you if it's safe to drink alcohol and how much is okay for you.

**What if I am pregnant or breast feeding?**

It's very important to talk to your doctor right away if you are pregnant, planning to get pregnant, or breastfeeding while taking rivaroxaban. Rivaroxaban might not be safe for your baby during pregnancy or while breastfeeding. Your doctor will help you decide on the safest plan for you and your baby. They might need to change your medicine or find another way to prevent blood clots during this time. Your doctor's goal is to keep both you and your baby healthy!

**Will I need to stop the medicine before surgery or dental procedures?**

Yes, you will likely need to stop taking rivaroxaban before surgery or certain dental procedures. Because rivaroxaban makes it easier to bleed, stopping it for a short time helps lower the risk of bleeding during the procedure. Your doctor will tell you exactly when to stop and when to restart rivaroxaban. Make sure your surgeon or dentist knows you're taking rivaroxaban, so they can talk to your doctor and plan things safely.

**Edoxaban**

**How does edoxaban work?**

Edoxaban is a medicine that helps keep your blood from clotting too much. It's like a helper that thins your blood a little. Your blood has special helpers that make clots, like tiny builders. Edoxaban blocks one of those helpers, called Factor Xa. By blocking Factor Xa, edoxaban makes it harder for clots to form. This helps prevent dangerous clots from happening. Take it exactly as your doctor tells you, and ask them or your pharmacist if you have any questions.

**How should I take it?**

Take edoxaban exactly as your doctor tells you, once a day. Swallow the tablet whole with water, without crushing or chewing, and you can take it with or without food. Try to take it at the same time each day to help you remember. If you miss a dose and it's the same day, take it; otherwise skip it and don't double up. Keep taking it even if you feel well unless your doctor says to stop. If you have any questions, ask your doctor or pharmacist.

**Do I need regular blood tests?**

Usually, you **don't need regular blood tests** while taking edoxaban, like people taking warfarin do. That's a nice thing about it! But your doctor might still want to check your blood sometimes, especially if you have kidney problems or if they're worried about bleeding. It's important to keep going to your checkups, so your doctor can make sure the medicine is working safely for you.

**Will the dose need to be adjusted?**

Yes, your doctor might need to change your dose of edoxaban sometimes. This could happen if you have kidney problems, because your kidneys help get the medicine out of your body. Also, certain other medicines can change how edoxaban works, so your doctor might need to adjust your dose if you're taking those too. It's important to tell your doctor about all the medicines you take, so they can make sure your edoxaban dose is just right.

**What will happen if I stop taking it?**

If you stop taking edoxaban without talking to your doctor first, your blood could start to clot too much again. Edoxaban helps prevent dangerous blood clots, so stopping it could put you at risk for problems like a stroke. It's important to talk to your doctor before you stop taking edoxaban, so they can tell you if it's safe and help you find another way to prevent blood clots if needed. Always check with your doctor before stopping.

**What are the potential side effects?**

Edoxaban can sometimes cause side effects, but most people don't have serious problems. The most common side effect is bleeding more easily. This might mean nosebleeds, bruising more easily, or bleeding gums when you brush your teeth.

If you notice any unusual bleeding or other new problems, tell your doctor right away. They can help you manage any side effects and make sure you're safe. Remember, the good things about taking edoxaban usually outweigh the risks of side effects.

**Do I need to do anything special about my diet while taking it?**

Good news! You usually don't need to change your diet while taking edoxaban. It's not affected by vitamin K in foods like leafy green vegetables. So just eat a healthy, balanced diet like you normally would. If you have any questions about your diet and edoxaban, talk to your doctor or pharmacist. They can give you advice that's just right for you.

If you have any questions about your diet and apixaban, talk to your doctor or pharmacist. They can give you advice that's just right for you.

**Is it safe to take other medications?**

It's really important to tell your doctor about all the other medicines you take, even over-the-counter ones like pain relievers, and also vitamins and supplements. Some medicines can change how edoxaban works in your body, making it too strong or not strong enough. Your doctor needs to know everything you're taking to keep you safe and make sure your medicines work well together. Never start or stop a medicine without talking to your doctor first!

**Can I drink alcohol while taking it? If yes, how much is safe to take?**

It's a good idea to talk to your doctor about drinking alcohol while you're taking edoxaban. Alcohol can sometimes increase your risk of bleeding, and edoxaban also makes you bleed more easily. So, drinking alcohol while taking edoxaban might make that risk bigger. Your doctor knows your health best and can tell you if it's safe to drink alcohol and how much is okay for you.

**What if I am pregnant or breast feeding?**

It's very important to talk to your doctor right away if you are pregnant, planning to get pregnant, or breastfeeding while taking edoxaban. Edoxaban might not be safe for your baby during pregnancy or while breastfeeding. Your doctor will help you decide on the safest plan for you and your baby. They might need to change your medicine or find another way to prevent blood clots during this time. Your doctor's goal is to keep both you and your baby healthy!

**Will I need to stop the medicine before surgery or dental procedures?**

Yes, you will likely need to stop taking edoxaban before surgery or certain dental procedures. Because edoxaban makes it easier to bleed, stopping it for a short time helps lower the risk of bleeding during the procedure. Your doctor will tell you exactly when to stop and when to restart edoxaban. Make sure your surgeon or dentist knows you're taking edoxaban, so they can talk to your doctor and plan things safely.

**ANNEXURE -D: Standard Responses**

**Warfarin**

**How does warfarin work?**

The formation of a blood clot in the body is a complex process that involves multiple substances called clotting factors. Warfarin decreases the body’s ability to form blood clots by blocking the formation of vitamin K–dependent clotting factors. Vitamin K is needed to make clotting factors and prevent bleeding. By giving a medication that blocks the clotting factors, your body can stop harmful clots from forming and prevent clots from getting larger.

**How should I take it?**

[Warfarin](https://www.uptodate.com/contents/warfarin-patient-drug-information?search=warfarin&topicRef=689&source=see_link) should be taken as directed, at approximately the same time each day. Often people take it at dinner time or in the evening, so that any instructions to change the dose can be followed. However, you should take your warfarin at the time that makes you most likely to take it consistently.

If a dose is missed or forgotten, take the missed dose as soon as possible, as long as it is within eight hours of the usual dosing time. If more than eight hours from the usual dosing time have passed, wait and take the next dose at the usual dosing time and let the health care team know that a dose was missed.

**Do I need regular blood tests?**

The blood test is called an INR test. The INR test checks to see how fast your blood clots. Your healthcare provider will decide what INR numbers are best for you. Your dose of warfarin will be adjusted to keep your INR in a target range for you. The target INR range depends upon the clinical situation. In most cases the target INR range will be between 2 and 3.

**Will the dose need to be adjusted?**

The dose of warfarin is adjusted to get the INR blood test into the correct range. The prothrombin time/international normalized ratio (PT/INR) is monitored more often when the dose is being changed, when the person starts or stops another medication, or when their medical condition changes. It is monitored less often when the dose is stable. A typical frequency of monitoring for stable dosing is approximately every four or six weeks.

**What will happen if I stop taking it?**

If you stop taking warfarin, your blood will start clotting at the same rate as before you started taking it, usually within a day or two of stopping. This means you may be at an increased risk of serious problems like strokes, heart attacks, deep vein thrombosis (DVT) or pulmonary embolism.

**What are the potential side effects?**

The major complication associated with warfarin is bleeding. This includes serious, life-threatening bleeding such as bleeding into the brain or internal bleeding, which is rare, and minor bleeding such as easy bruising, gum bleeding, or nosebleeds, which are common and can occur with any anticoagulant. Warfarin can also cause a rare side effect called skin necrosis or gangrene, which can cause dark red or black areas on the skin.

**Do I need to do anything special about my diet while taking it?**

**Vitamin K** – The use of [warfarin](https://www.uptodate.com/contents/warfarin-patient-drug-information?search=warfarin&topicRef=689&source=see_link) should not interfere with a healthy diet. However, it is ideal not to have large weekly variations in the amount of vitamin K intake from food, because changes in the weekly amount of vitamin K intake can alter the international normalized ratio (INR). People who take warfarin should aim to eat a relatively similar amount of vitamin K-containing foods each week. Moderate daily intake of approximately the same amount of vitamin K is likely to keep the INR more stable. Switching to a lower amount of vitamin K can increase the INR, potentially increasing the risk of bleeding, and switching to a greater amount of vitamin K can lower the INR, potentially increasing the risk of blood clots. Some foods that have a high level of vitamin K include: kale, broccoli, spinach, collard or turnip greens, lettuce, Brussels sprouts, and cabbage.

**Cranberry juice and grapefruit juice** – There have been mixed reports on the effect of cranberry juice or grapefruit juice in people who use [warfarin](https://www.uptodate.com/contents/warfarin-patient-drug-information?search=warfarin&topicRef=689&source=see_link) to prevent blood clots. Some clinicians suggest that people taking warfarin not consume more than one or two glasses of either juice per day.

**Calcium supplements** – Some calcium supplements contain vitamin K. Make sure to check the ingredients before starting a new calcium supplement.

**Is it safe to take other medications?**

Many medicines can affect the dose of warfarin needed. Talk to your doctor if you are prescribed a new medicine. Avoid aspirin and "NSAID" medicines. Most people can use acetaminophen (paracetamol) for pain or fever. medicines for heart problems, such as amiodarone, quinidine or propafenone. tramadol, a strong painkiller. cholesterol-lowering medicines, such as bezafibrate, gemfibrozil, clofibrate or cholestyramine. antibiotics, such as erythromycin, co-trimoxazole or norfloxacin.

Many herbs, including ginkgo, green tea, and garlic pills, could increase the risk of bleeding with anticoagulant medicines. Avoid St. John's Wort.

**Can I drink alcohol while taking it? If yes, how much is safe to take?**

Alcohol in low or moderate amounts (one or two servings per day) is unlikely to have major effects on the INR. People who consume alcohol should limit intake to one to two servings per day. A serving is equal to 1 beer (12 ounces), 1 glass of wine (5 ounces), or 1.5 ounces of spirits.

People on [warfarin](https://www.uptodate.com/contents/warfarin-patient-drug-information?search=warfarin&topicRef=689&source=see_link) therapy should avoid drinking excessive amounts of alcohol over a short period of time (e.g., with a single meal) because this can affect the INR and increase the risk of injury and serious bleeding.

**What if I am pregnant or breast feeding?**

Warfarin is not recommended during pregnancy as it can sometimes cause birth defects, miscarriage, or death of your unborn baby.

Warfarin is considered safe for use in breastfeeding. speak to the health care team if you plan to breastfeed.

**Will I need to stop the medicine before surgery or dental procedures?**

Because of the risk of bleeding, your dose of warfarin may need to be lowered or stopped a few days before you have an operation. Most people are switched to a different medicine that comes as a shot. Tell the surgeon or nurse that you're taking warfarin. Most people do not need to change their warfarin dose for minor dental procedures.

**Apixaban**

**How does apixaban work?**

Many things must happen inside the body for a blood cloth to form. Natural parts of a blood called clotting factors are involved in this process. Apixaban blocks one of the clotting factors, factor Xa. Apixaban can help stop clots from forming and can keep clots from getting bigger, but it does not break up clots that already exist.

**How should I take it?**

it is Once or twice a day, Swallow the tablet with a drink of water. Apixaban can be taken with or without food. Some people might need to take a higher dose twice a day for the first week, then switch to a lower dose twice a day. If you have trouble swallowing this drug, it can be crushed and mixed in water, apple juice, or applesauce. If you crush and mix this drug, take it within 4 hours of mixing.

 Take a missed dose as soon as you think about it. If your next dose is 6 or more hours away, take the missed dose as soon as you remember. If your next dose is less than 6 hours away, skip the missed dose.  if ifif

**Do I need regular blood tests?**

No, apixaban does not need regular blood tests to monitor your clotting levels (INR test). However, you should have a blood test to monitor your kidney and liver function before you start taking apixaban. Further blood tests may be required if your doctor feels they are necessary.

**Will the dose need to be adjusted?**

No. Once you are on a stable dose, you do not need to adjust the dose.

**What will happen if I stop taking it?**

If you stop taking it, your blood will start clotting at the same rate as before you started taking it, usually within a day or two of stopping. This means you may be at an increased risk of serious problems like strokes, heart attacks, deep vein thrombosis (DVT) or pulmonary embolism.

**What are the potential side effects?**

Contact your doctor immediately, or go to your nearest hospital emergency department if you:

- Signs of an allergic reaction, like rash; hives; itching; red, swollen, blistered, or peeling skin with or without fever; wheezing; tightness in the chest or throat; trouble breathing, swallowing, or talking; unusual hoarseness; or swelling of the mouth, face, lips, tongue, or throat.
- Signs of bleeding like throwing up or coughing up blood; vomit that looks like coffee grounds; blood in the urine; black, red, or tarry stools; bleeding from the gums; abnormal vaginal bleeding; bruises without a cause or that get bigger; or bleeding you cannot stop.
- Weakness on 1 side of the body, trouble speaking or thinking, change in balance, drooping on one side of the face, or blurred eyesight.
- Dizziness or passing out.
- Feeling tired or weak.
- Feeling confused.
- Joint pain or swelling.
- Chest pain or pressure.

**Do I need to do anything special about my diet while taking it?**

You need to avoid grapefruit and grapefruit juice, which can change the way the medicine works.

**Is it safe to take other medications?**

A few medicines can affect the dose of the anticoagulant.

- Avoid aspirin and "NSAID" medicines unless advised by your doctor. Most people can use acetaminophen (paracetamol) for pain or fever.
- Many herbs, including ginkgo, green tea, and garlic pills, could increase the risk of bleeding with anticoagulant medicines.
- Avoid St. John's Wort.

**Can I drink alcohol while taking it? If yes, how much is safe to take?**

It is best to avoid alcohol while taking apixaban. Alcohol can increase the risk of bleeding in your stomach and intestines.

If you do drink alcohol, do not have more than 1 drink a day.

One drink = 1 beer (12 oz) or 1 glass of wine (4 oz) or 1 cocktail (2 oz) or 1 shot (1.5 oz).

**What if I am pregnant or breast feeding?**

Apixaban should not be taken during pregnancy. If you take apixaban and are planning a pregnancy or you become pregnant while taking it, speak to your doctor or specialist. They will change you to a different medicine.

Most people are switched to a different medicine that comes as a shot.

Apixaban is not usually recommended while breastfeeding.

**Will I need to stop the medicine before surgery or dental procedures?**

Your doctor may tell you to stop apixaban for 1 or more days before medical or dental procedures, including surgery. You must stop apixaban before any procedure that uses an epidural catheter.

**Dabigatran**

**How does dabigatran work?**

Dabigatran works by stopping a clotting factor called thrombin from working. This thins your blood so it takes longer to clot.

**How should I take it?**

it is Once or twice a day with or without food, take with full glass of water, take with food if it causes an upset stomach. If you are taking 2 doses in a day, take doses about 12 hours apart. Swallow whole. Do not chew, open, or crush. Keep taking this drug as you have been told by your doctor or other health care provider, even if you feel well.

Take a missed dose as soon as you think about it. If it is less than 6 hours until the next dose, skip the missed dose and go back to the normal time. Do not take 2 doses at the same time or extra doses.

**Do I need regular blood tests?**

No, dabigatran does not need regular blood tests to monitor your clotting levels (INR test). However, you should have a blood test to monitor your kidney and liver function before you start taking dabigatran. Further blood tests may be required if your doctor feels they are necessary.

**Will the dose need to be adjusted?**

No. Once you are on a stable dose, you do not need to adjust the dose.

**What will happen if I stop taking it?**

If you stop taking it, your blood will start clotting at the same rate as before you started taking it, usually within a day or two of stopping. This means you may be at an increased risk of serious problems like strokes, heart attacks, deep vein thrombosis (DVT) or pulmonary embolism.

**What are the potential side effects?**

Contact your doctor immediately, or go to your nearest hospital emergency department if you:

- have a nose bleed
- have red or dark urine
- start coughing up blood
- vomit, and it is dark or blood stained
- have red or black (tar like) bowel motions
- have excessive bleeding, or bleeding that lasts longer than usual
- have unexplained bruising, or bruising that gets bigger without a cause
- have a bad headache or dizziness

**Other side effects of DABIGATRAN can include**: heartburn, indigestion nausea, vomiting, diarrhoea, stomach ache, dizziness, breathlessness, fever, yellowing of skin and/or eyes (jaundice).

**Do I need to do anything special about my diet while taking it?**

No. There are no dietary restrictions while taking dabigatran.

**Is it safe to take other medications?**

A few medicines can affect the dose of the anticoagulant. Avoid aspirin and "NSAID" medicines unless advised by your doctor. Most people can use acetaminophen (paracetamol) for pain or fever.

Tell your doctor if you're taking any of these medicines before you start taking dabigatran:

- any other anticoagulant, such as warfarin or enoxaparin, any other medicines that affect blood clotting, such as clopidogrel, ticagrelor or prasugrel.
- medicines to treat heart problems, such as verapamil, amiodarone or dronedarone.
- medicines to treat fungal or bacterial infections, such as fluconazole, ketoconazole or clarithromycin.
- medicines to treat depression, such as fluoxetine, sertraline or citalopram.
- medicines to treat HIV, such as ritonavir.
- medicines to treat epilepsy, such as carbamazepine or phenytoin.
- non-steroidal anti-inflammatory drugs (NSAIDs), such as ibuprofen or aspirin.

Many herbs, including ginkgo, green tea, and garlic pills, could increase the risk of bleeding with anticoagulant medicines. Avoid St. John's Wort.

**Can I drink alcohol while taking it? If yes, how much is safe to take?**

It is best to avoid alcohol while taking dabigatran. Alcohol can increase the risk of bleeding in your stomach and intestines. If you do drink alcohol, do not have more than 1 drink a day.

One drink = 1 beer (12 oz) or 1 glass of wine (4 oz) or 1 cocktail (2 oz) or 1 shot (1.5 oz).

**What if I am pregnant or breast feeding?**

Dabigatran should not be taken during pregnancy. If you take dabigatran and are planning a pregnancy or you become pregnant while taking it, speak to your doctor or specialist. They will change you to a different medicine.

It may be OK to take dabigatran while breastfeeding, but talk to your doctor or pharmacist first.

**Will I need to stop the medicine before surgery or dental procedures?**

Because of the risk of bleeding, your dose of Dabigatran may need to be lowered or stopped a few days before you have an operation.

Most people are switched to a different medicine that comes as a shot.

**Rivaroxaban**

**How does rivaroxaban work?**

Rivaroxaban works by stopping a clotting factor called factor Xa from working. This thins your blood so it takes longer to clot.

**How should I take it?**

You'll usually take rivaroxaban once a day just after a meal or snack. It's important to take it with some food to help your body absorb the whole dose. Try to take it at the same time every day. Some people might need to take a lower dose twice a day for the first 3 weeks, then switch to a higher dose once a day.

- If you take rivaroxaban once a day, take the missed dose as soon as you remember it on that day. Resume your regular dosing schedule the next day.
- If you take rivaroxaban twice a day for the treatment of a Deep vein thrombosis (DVT) or pulmonary embolism (PE), take the missed dose as soon as you remember it on that day. You may take 2 doses at the same time to make up for the missed dose. Resume your regular dosing schedule on the next day.
- If you have coronary artery disease (CAD) or peripheral artery disease (PAD) and take rivaroxaban twice a day to reduce the risk of DVT and PE and miss a dose, just continue your regular dosing schedule. Do not take a double dose to make up for a missed one.

**Do I need regular blood tests?**

No, rivaroxaban does not need regular blood tests to monitor your clotting levels (INR test). However, you should have a blood test to monitor your kidney and liver function before you start taking rivaroxaban. Further blood tests may be required if your doctor feels they are necessary.

**Will the dose need to be adjusted?**

No. Once you are on a stable dose, you do not need to adjust the dose.

**What will happen if I stop taking it?**

If you stop taking it, your blood will start clotting at the same rate as before you started taking it, usually within a day or two of stopping. This means you may be at an increased risk of serious problems like strokes, heart attacks, deep vein thrombosis (DVT) or pulmonary embolism.

**What are the potential side effects?**

Bleeding is the main side effect of RIVAROXABAN. To lower the chance of bleeding, take your medicine exactly as your doctor explained.

Contact your doctor immediately, or go to your nearest hospital emergency department if you:

- have a nose bleed
- have red or dark urine
- start coughing up blood
- vomit, and it is dark or blood stained
- have red or black (tar like) bowel motions
- have excessive bleeding, or bleeding that lasts longer than usual
- have unexplained bruising, or bruising that gets bigger without a cause
- have a bad headache or dizziness

Other side effects of RIVAROXABAN can include: heartburn, indigestion, nausea, vomiting, diarrhoea, stomach ache, dizziness, breathlessness, fever, yellowing of skin and/or eyes (jaundice).

**Do I need to do anything special about my diet while taking it?**

You need to avoid grapefruit and grapefruit juice, which can change the way the medicine works. If you take rivaroxaban for atrial fibrillation ("A-fib"), you need to take it with your evening meal. If you take rivaroxaban (for any reason) and the strength of your pills is 15 or 20 mg, you need to take it with food.

**Is it safe to take other medications?**

A few medicines can affect the dose of the anticoagulant. Avoid aspirin and "NSAID" medicines unless advised by your doctor. Most people can use acetaminophen (paracetamol) for pain or fever.

Many herbs, including ginkgo, green tea, and garlic pills, could increase the risk of bleeding with anticoagulant medicines. Avoid St. John's Wort.

**Can I drink alcohol while taking it? If yes, how much is safe to take?**

It is best to avoid alcohol while taking rivaroxaban. Alcohol can increase the risk of bleeding in your stomach and intestines. If you do drink alcohol, do not have more than 1 drink a day.

One drink = 1 beer (12 oz) or 1 glass of wine (4 oz) or 1 cocktail (2 oz) or 1 shot (1.5 oz).

**What if I am pregnant or breast feeding?**

RIVAROXABAN is NOT recommended during pregnancy or whilst breastfeeding. If you plan to become pregnant or think you are pregnant, tell your doctor as soon as possible.

**Will I need to stop the medicine before surgery or dental procedures?**

Rivaroxaban may need to be stopped for one or more days before any surgery or medical or dental procedure. Your doctor will tell you when to stop taking rivaroxaban and when to start taking rivaroxaban again after your surgery or procedure. Most people are switched to a different medicine that comes as a shot.

**Edoxaban**

**How does edoxaban work?**

Edoxaban is an anticoagulant medicine that helps to prevent blood from clotting as quickly as normal. It does this by interfering with a substance in the body called Factor Xa which is involved in the development of blood clots.

**How should I take it?**

You can take edoxaban with or without food, preferably with water, but try to take it at the same time every day. It is important not to skip doses. If a dose is missed the patient should take edoxaban immediately and continue on the following day with the once daily intake as recommended. The dose should not be doubled within the same day to make up for a missed dose.

**Do I need regular blood tests?**

No, Edoxaban does not need regular blood tests to monitor your clotting levels (INR test). However, you should have a blood test to monitor your kidney and liver function before you start taking Edoxaban. Further blood tests may be required if your doctor feels they are necessary.

**Will the dose need to be adjusted?**

No. Once you are on a stable dose, you do not need to adjust the dose.

**What will happen if I stop taking it?**

If you stop taking it, your blood will start clotting at the same rate as before you started taking it, usually within a day or two of stopping. This means you may be at an increased risk of serious problems like strokes, heart attacks, deep vein thrombosis (DVT) or pulmonary embolism.

**What are the potential side effects?**

As this medicine affects blood clotting, most side effects are related to signs such as bruising or bleeding. Tell your doctor immediately if you experience any of the following side effects: Long or excessive bleeding, exceptional weakness, tiredness, paleness, dizziness, headache or unexplained swelling. Also report if there is any blood in the urine or passing black tarry or blood-stained stools. Patients should seek urgent medical attention if they fall or injure themselves during treatment, especially if they hit their head, due to the increased risk of bleeding.

Common side effects in people who take edoxaban is low red blood cell count (anaemia) Talk to your doctor if you have any side effect that bothers you or that does not go away.

**Do I need to do anything special about my diet while taking it?**

No. There are no dietary restrictions while taking edoxaban.

**Is it safe to take other medications?**

A few medicines can affect the dose of the anticoagulant.

- Avoid aspirin and "NSAID" medicines unless advised by your doctor. Most people can use acetaminophen (paracetamol) for pain or fever.
- Many herbs, including ginkgo, green tea, and garlic pills, could increase the risk of bleeding with anticoagulant medicines.
- Avoid St. John's Wort.
- Paracetamol based pain killers are acceptable.

**Can I drink alcohol while taking it? If yes, how much is safe to take?**

It is best to avoid alcohol while taking apixaban. Alcohol can increase the risk of bleeding in your stomach and intestines. If you do drink alcohol, do not have more than 1 drink a day.

One drink = 1 beer (12 oz) or 1 glass of wine (4 oz) or 1 cocktail (2 oz) or 1 shot (1.5 oz).

**What if I am pregnant or breast feeding?**

Edoxaban should not be taken during pregnancy. If you take dabigatran and are planning a pregnancy or you become pregnant while taking it, speak to your doctor or specialist. They will change you to a different medicine.

Edoxaban is not usually recommended while breastfeeding as there is not enough information to show that it's safe.

**Will I need to stop the medicine before surgery or dental procedures?**

Patients should not stop taking edoxaban without first talking to their doctor or dentist. Edoxaban may need to be stopped for one or more days before any planned surgery, dental or medical procedure. Most people are switched to a different medicine that comes as a shot.

Most people do not need to change their edoxaban dose but your dentist may contact your doctor for advice before starting the dental treatment.
